# A Role for Myosin VI in the Localization of Axonal Proteins

**DOI:** 10.1371/journal.pbio.1001021

**Published:** 2011-03-01

**Authors:** Tommy L. Lewis, Tianyi Mao, Don B. Arnold

**Affiliations:** 1 Department of Biological Sciences, University of Southern California, Los Angeles, California, United States of America; 2 Program in Molecular and Computational Biology, University of Southern California, Los Angeles, California, United States of America; 3 Janelia Farm Research Campus, Howard Hughes Medical Institute, Ashburn, Virginia, United States of America; University of Cambridge, United Kingdom

## Abstract

In neurons polarized trafficking of vesicle-bound membrane proteins gives rise to the distinct molecular composition and functional properties of axons and dendrites. Despite their central role in shaping neuronal form and function, surprisingly little is known about the molecular processes that mediate polarized targeting of neuronal proteins. Recently, the plus-end-directed motor Myosin Va was shown to play a critical role in targeting of transmembrane proteins to dendrites; however, the role of myosin motors in axonal targeting is unknown. Here we show that Myosin VI, a minus-end-directed motor, plays a vital role in the enrichment of proteins on the surface of axons. Engineering non-neuronal proteins to interact with Myosin VI causes them to become highly concentrated at the axonal surface in dissociated rat cortical neurons. Furthermore, disruption of either Myosin VI function or expression leads to aberrant dendritic localization of axonal proteins. Myosin VI mediates the enrichment of proteins on the axonal surface at least in part by stimulating dendrite-specific endocytosis, a mechanism that has been shown to underlie the localization of many axonal proteins. In addition, a version of Channelrhodopsin 2 that was engineered to bind to Myosin VI is concentrated at the surface of the axon of cortical neurons in mice in vivo, suggesting that it could be a useful tool for probing circuit structure and function. Together, our results indicate that myosins help shape the polarized distributions of both axonal and dendritic proteins.

## Author Summary

Following synthesis in the endoplasmic reticulum (ER) and Golgi apparatus, neuronal proteins follow divergent trafficking pathways to the axonal and dendritic plasma membranes. This specialized trafficking depends on motor proteins that move along microtubules or actin in either a “plus-end” or “minus-end” direction. Although the molecular details of these pathways are poorly understood, recent work suggests that a plus-end-directed myosin motor guides proteins preferentially to dendrites. Here we find that Myosin VI, a minus-end-directed motor, plays a role in the concentration of proteins at the surface of the axon. Several studies have shown that many axonal proteins are targeted to both compartments initially, and are subsequently enriched on the axonal surface after they have been specifically removed from the surface of the dendrites by endocytosis. We show here that this dendrite-specific endocytosis is promoted by interaction with Myosin VI, whereas blocking Myosin VI function prevents axonal protein from being internalized from the surface of dendrites. Our results suggest a model where neuronal proteins are enriched on the surface of either axons or dendrites based on the properties of the myosin motor with which they interact.

## Introduction

Following synthesis in the secretory pathway, axonal and dendritic transmembrane proteins follow distinct transport pathways to the plasma membrane. After being sorted into separate sets of vesicles at the trans-Golgi membrane [1], dendritic proteins are transported directly to the somatodendritic domain without entering the distal axonal domain, whereas axonal proteins enter both axons and dendrites [2]–[4]. Axonal proteins that are carried to the dendrites are unloaded onto the plasma membrane and subsequently reloaded into vesicles that travel to the axonal membrane [4],[5]. The net effect of these processes is that axonal transmembrane proteins are distributed roughly evenly to the intracellular regions of both the axon and dendrites, but are dramatically enriched on the axonal membrane.

The transport of both axonal and dendritic proteins is mediated by kinesin motors [6]–[10]. However, targeting of dendritic proteins cannot be explained by the intrinsic properties of dendritic kinesins, such as Kif17, which cannot autonomously distinguish between axonal and dendritic microtubules [11]. Instead, recent work from our laboratory indicates that vesicles are targeted to dendrites through the actions of plus-end-directed myosin motors, which direct the vesicles away from the axon and towards the cell body [12]. This result suggests that a minus-end-directed motor might participate in the localization of axonal proteins. Interestingly, of the 35 known classes of myosin motors, members of only one, Myosin VI, are known to move towards the pointed (or minus) end of actin filament [13],. The involvement of Myosin VI in endocytosis further suggests that it might contribute to the enrichment of proteins on the surface of the axon. It associates with both clathrin-coated vesicles and proteins that mediate endocytosis [15]–[17] and plays a prominent role in the endocytosis of at least two neuronal proteins [18],[19].

Here, we show that Myosin VI [20] plays a role in the concentration of proteins at the axonal surface of neurons in dissociated cultures. Myosin VI mediates this axonal enrichment at least in part by enhancing dendrite-specific endocytosis. In addition, we find evidence that is consistent with Myosin VI promoting the direct trafficking of proteins to the axon. Thus, myosin motors contribute to the enrichment of both axonal and dendritic proteins at the plasma membranes of the appropriate polar compartments. In this manner they play a role in specifying the polarized structure of neurons, which is necessary for proper electrical signaling.

## Results

### Interaction with Myosin VI Promotes Enrichment of Proteins at the Axonal Surface

**Figure 1:**
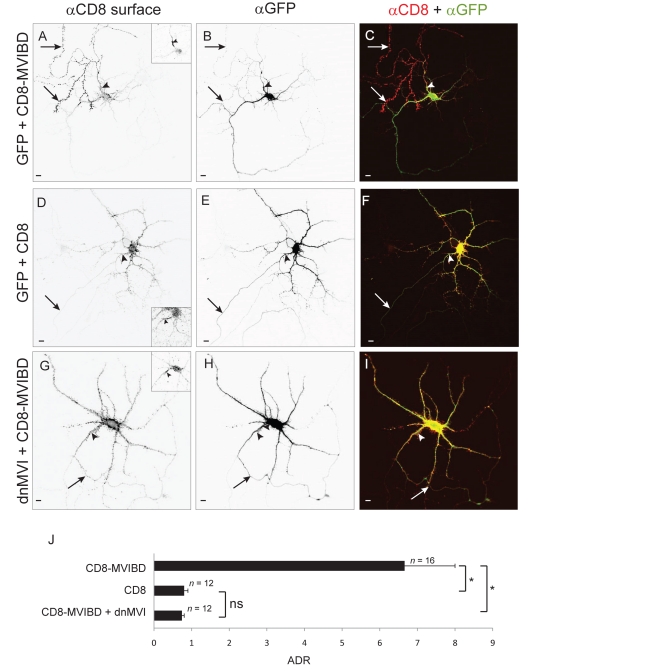
Interaction with Myosin VI is sufficient to enrich a heterologous protein at the surface of the axon. In a cortical neuron in dissociated culture, surface-labeled CD8 fused to MVIBD (CD8-MVIBD) (A) is highly enriched at the surface of the axon, in contrast to the nonspecific localization of coexpressed GFP (B). (C) Merge of CD8-MVIBD (red) and GFP (green). In contrast, when CD8 (D) is coexpressed with GFP (E), it localizes nonspecifically. (F) Merge of CD8 (red) and GFP (green) showing that the two proteins colocalize. When CD8-MVIBD (G) is coexpressed with GFP-dnMVI (H), it localizes nonspecifically. (I) Merge of CD8-MVIBD (red) and GFP-dnMVI (green) indicates that the two proteins colocalize. (J) The average ADR of CD8-MVIBD is greater than 6-fold higher than that of CD8 when coexpressed with GFP or of CD8-MVIBD when coexpressed with dnMVI. *, *p*<0.0001; ns, *p*>0.5, Wilcoxon-Mann-Whitney test. Insets show staining of endogenous Ankyrin G. Arrows point to the axon; arrowheads point to the axon initial segment. Scale bars are 10 µm.

In a previous study we found that by engineering non-neuronal proteins to interact with Myosin Va, a plus-end-directed motor, we could target the proteins specifically to the somatodendritic compartment. To determine whether, conversely, interaction with the only known minus-end-directed motor, Myosin VI (Figure S1), could direct proteins to the surface of the axon, we fused a Myosin VI binding domain (MVIBD; Figure S2) onto the C-terminus of the non-neuronal protein CD8 (to give CD8-MVIBD). MVIBD consists of two known Myosin VI binding sites fused in tandem, one from Optineurin, a protein that is associated with amyotrophic lateral sclerosis [21] and a second from Disabled homolog 2 (DAB2), which has been associated with prostate cancer [22]. Because each binding site interacts with a distinct region of Myosin VI, we reasoned putting the two sites in tandem would create a binding site with a greater affinity for Myosin VI than that of either domain alone. Strikingly, when expressed in cortical neurons in dissociated culture, CD8-MVIBD was highly enriched on the surface of the axon as compared with the surface of the soma and dendrites (Figure 1A–1C). To complement these qualitative observations we calculated the axon to dendrite ratio (ADR) [12], which is a relative measure of the concentration of protein at the surface of the axon versus the dendrites. For CD8-MVIBD the ADR of 6.6±1.3 (*n = *16) is consistent with enrichment in the axon. This is significantly different from the ADR of CD8 (Figure 1D and 1F; 0.8±0.1, *n = *12, *p*<0.0001). To determine whether this enrichment takes place through interaction with Myosin VI, we first blocked Myosin VI function using a dominant negative construct consisting of its tail domain (dominant negative Myosin VI [dnMVI]). When coexpressed with green fluorescent protein (GFP)–dnMVI, CD8-MVIBD localized nonspecifically, with an ADR of 0.79±0.07 (*n = *12; Figure 1G–1I), which is significantly different from the ADR of CD8-MVIBD coexpressed with GFP (*p*<0.0001). We also tested the effect of reducing Myosin VI levels by co-transfecting a short interfering RNA (siRNA) against Myosin VI (Figure S3) along with CD8-MVIBD. Under these conditions CD8-MVIBD localized nonspecifically (ADR  = 0.98±0.15, *n = *12), which is significantly different from the ADR of CD8-MVIBD coexpressed with an empty siRNA vector (ADR  = 4.0±0.3, *n = *12, *p*<0.0001; Figure S4). Thus, interaction with Myosin VI can promote the enrichment of a non-neuronal transmembrane protein at the surface of the axon.

### Myosin VI Is Necessary for Enrichment of Proteins at the Axonal Surface

**Figure 2:**
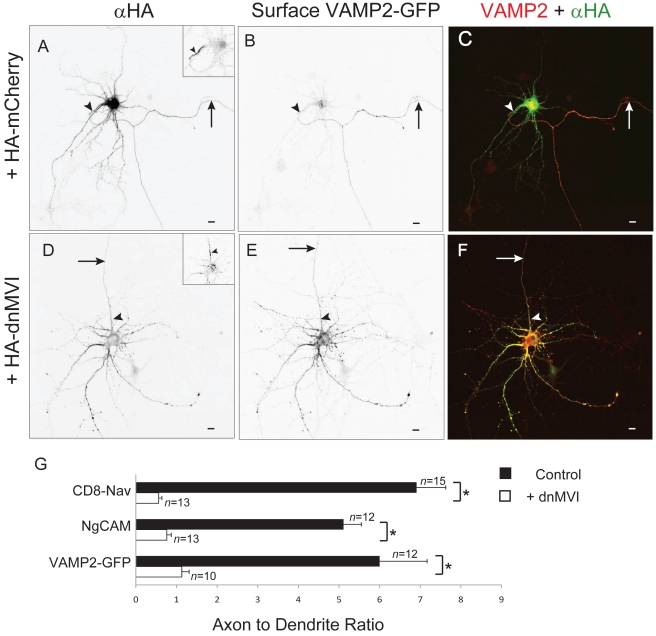
Myosin VI function is necessary for enrichment of proteins at the axonal surface. In a cortical neuron expressing exogenous HA-mCherry (A) and VAMP2-GFP (B), surface VAMP2-GFP localized specifically to the axon. (C) Merge of surface VAMP2-GFP (red) and HA-mCherry (green). In contrast, when expressed with HA-dnMVI (D), a dominant negative version of Myosin VI, surface VAMP2-GFP (E) localized nonspecifically to both axons and dendrites. (F) Merge of surface VAMP2-GFP (red) and HA-dnMVI (green). (G) The ADRs (see Materials and Methods) of exogenous surface CD8-Nav, NgCAM, and VAMP2-GFP coexpressed with either GFP- or HA-tagged dnMVI (white bars), are 5- to 6-fold lower than when each is coexpressed with either GFP or HA-mCherry (black bars). Insets show staining of endogenous Ankyrin G. Arrows point to the axon; arrowheads point to the axon initial segment. Scale bars are 10 µm. *, *p*<0.0003 (Wilcoxon-Mann-Whitney test).

**Figure 3:**
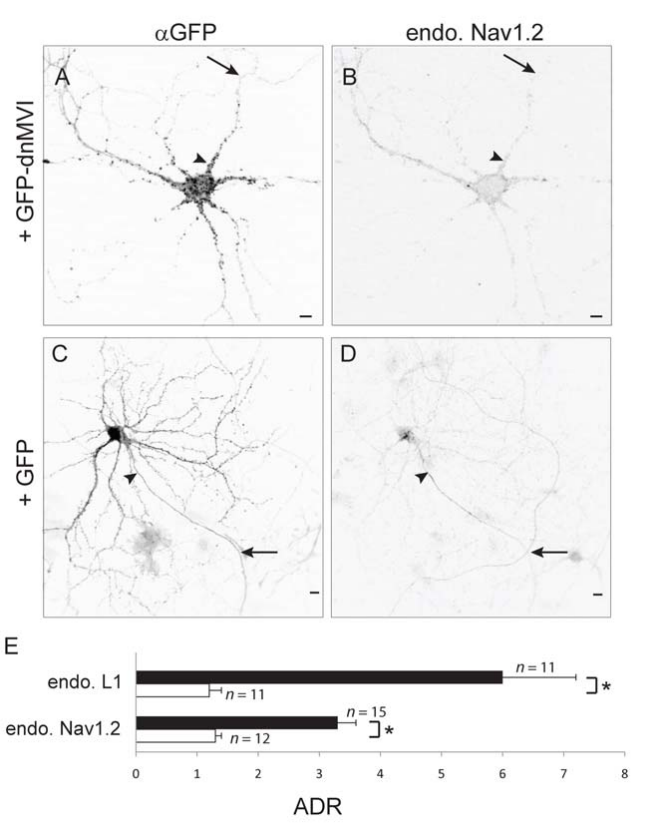
Blocking Myosin VI function blocks the concentration of endogenous Nav1.2 at the axon. In a cortical neuron transfected with GFP-dnMVI (A), endogenous Nav1.2 (B) localizes in both axons and dendrites in a nonspecific manner. In contrast, in a neuron expressing GFP (C), endogenous Nav1.2 (D) is localized specifically to the axon. (E) The ADRs of endogenous L1 and Nav1.2 were significantly higher when coexpressed with GFP (black bars) than when coexpressed with GFP dnMVI (white bars). *, *p*<0.0005, Wilcoxon-Mann-Whitney test. Arrows point to the axon; arrowheads point to the axon initial segment. Scale bars are 10 µm.

To further investigate a possible role for Myosin VI in the concentration of proteins at the axonal surface, we examined the effect of disrupting Myosin VI function on the distribution of native or introduced axonal proteins. For these experiments we chose to study the axonal proteins NgCAM, VAMP2-GFP, and CD8-Nav, each of which has been shown to localize to the axon in dissociated cultures [1],[3],[4]. Note that CD8-Nav is a fusion between CD8 and an axonal targeting motif from the sodium channel Nav1.2 [3]. Remarkably, while CD8-Nav, NgCAM, and VAMP2-GFP were each substantially more concentrated on the surface of axons than on the dendritic surface under control conditions (ADR  = 6.91±0.91, 5.11±0.76, 6.0±0.44; *n = *15, 12, 12, respectively; Figure 2A–C), blocking Myosin VI function by coexpression with dnMVI caused each to localize nonspecifically (ADR  = 0.61±0.10, 0.79±0.09, 1.1±0.4; *n = *13, 13, 10, respectively; *p*<0.0001 for each pair; Figures 2D–2G and S5). To determine whether Myosin VI is also necessary for the concentration of endogenous transmembrane proteins at the axonal surface, we examined the effect of blocking Myosin VI on localization of L1, the mammalian homolog of NgCAM and the sodium channel Nav1.2. In cells expressing GFP, endogenous L1 (ADR  = 6.0±1.2, *n = *11) and Nav1.2 (ADR  = 3.3±0.3, *n = *15) each were enriched at the axonal surface (Figures 3 and S5), whereas in cells expressing GFP-dnMVI, L1 and Nav1.2 were each present on the surfaces of both compartments at roughly equal concentrations (ADR  = 1.3±0.2, 1.24±0.1; *n = *11, 12; Figures 3 and S5). The ADRs for L1 and Nav1.2 differed significantly under the two conditions (*p*<0.0005 for both comparisons).

**Figure 4:**
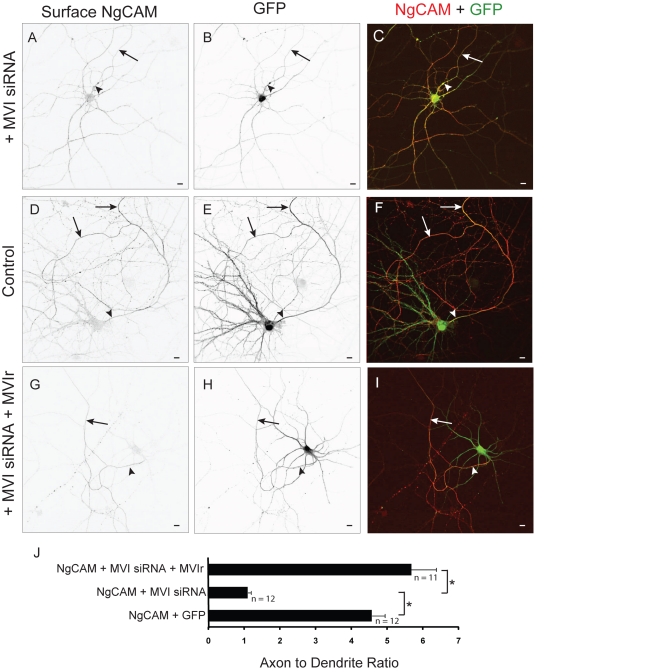
Knockdown of Myosin VI with siRNA blocks axonal enrichment of NgCAM. In a cortical neuron transfected with siRNA against Myosin VI (MVI siRNA), surface exogenous NgCAM (A) localized both to axons and to dendrites, with an expression pattern that overlaps that of GFP (B). (C) Merge of surface NgCAM (red) and GFP (green) in a cell coexpressing MVI siRNA. In contrast, NgCAM (D) was enriched on the surface of the axon when expressed with GFP (E). (F) Merge of surface NgCAM (red) and GFP (green). When expressed with both siRNA against Myosin VI and a version of Myosin VI that is impervious to siRNA (MVIr), surface NgCAM (G) localized specifically to the axon, in contrast to the nonspecific localization of GFP (H). (I) Merge of surface NgCAM (red) and GFP (green) in a cell cotransfected with MVI siRNA and MVIr. (J) When coexpressed with either GFP alone or with MVI siRNA and MVIr, NgCAM localized to the axon with an ADR 4- to 5-fold higher than when NgCAM was coexpressed with MVI siRNA alone. Arrows point to the axon; arrowheads point to the axon initial segment. Scale bars are 10 µm. *, *p*<0.0001 (Wilcoxon-Mann-Whitney test).

We also tested the effect of disrupting Myosin VI with siRNA (Figure S3) on the localization of NgCAM. NgCAM localized nonspecifically when expressed with Myosin VI siRNA (MVI siRNA) (ADR  = 1.1±0.1, *n = *12), a significant change in distribution as compared with the distribution of NgCAM coexpressed with an empty siRNA vector (ADR = 4.57±0.36; *p*<0.0001; Figure 4A–4F). Moreover, we were able to rescue the polarized distribution of NgCAM by coexpression of the MVI siRNA with a form of Myosin VI (Myosin VI rescue [MVIr]) that contains silent mutations that make it impervious to the MVI siRNA (ADR  = 5.67±0.7, *n = *11), a result that is significantly different (*p*<0.0001) from the ADR of NgCAM when transfected with siRNA alone (Figure 4A–4J).

**Figure 5:**
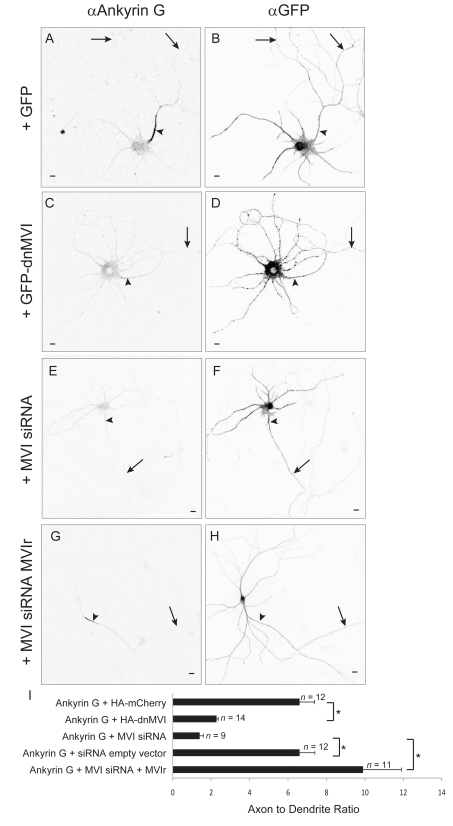
Myosin VI function is necessary for axonal enrichment of Ankyrin G. Endogenous Ankyrin G (A) localizes very specifically to the axon initial segment in neurons expressing GFP (B). In contrast, Ankyrin G (C) localizes in a relatively nonspecific manner in neurons expressing GFP-dnMVI (D). Similarly, endogenous Ankyrin G (E) localizes nonspecifically when coexpressed with MVI siRNA and GFP (F). In contrast, endogenous Ankyrin G (G) when coexpressed with MVIr and GFP (H) localizes specifically to the axon initial segment. (I) ADRs indicate that disrupting Myosin VI function or expression causes Ankyrin G to localize nonspecifically. *, *p*<0.0002. Arrows point to the axon; arrowheads point to the axon initial segment. Scale bars are 10 µm.

In the experiments described in Figure 2 all cells that were included exhibited polarized distributions of the axonal protein Ankyrin G, which was used to distinguish the axon from the dendrites (see Materials and Methods). For instance, in the experiments in which we coexpressed NgCAM with HA-dnMVI, Ankyrin G had an overall ADR of 6±1 (*n = *13). In general, however, the polarity of Ankyrin G was significantly reduced in cultures that expressed HA-dnMVI (Figure 5A–5D; ADR  = 2.28±0.09, *n = *14) versus HA-mCherry (ADR  = 9.7±0.65, *n = *12, *p*<0.0001). Expressing siRNA against Myosin VI also reduced the polarity of Ankyrin G (Figure 5E–5I; ADR  = 1.4±0.2, *n = *9) in comparison with an empty siRNA vector (ADR  = 6.6±1.2, *n = *12, *p*<0.0002) or with siRNA and the rescue construct MVIr (ADR  = 9.9±2, *n = *11, *p*<0.0001). Because the elimination of Ankyrin G has been associated with the loss of neuronal polarity [23], it is possible that axonal proteins became mislocalized when coexpressed with dnMVI or MVI siRNA because of a general loss of cell polarity. However, three observations make this unlikely. First, in cells expressing dnMVI and CD8-Nav, NgCAM, or VAMP2, the ADR of each in individual cells was completely independent of the degree of polarization of Ankyrin G in the same cells (Figure S6). Second, in dnMVI- or MVI siRNA-expressing cells where the localization of Ankyrin G was disrupted, dendritic proteins continued to be localized to the dendrite (Figure S7), indicating that the diffusion barrier was intact [24], and that overall cell polarity was not disrupted. Finally, siRNA against Myosin VI, which disrupted localization of Ankyrin G (Figure 5), did not disrupt the morphological polarity of cells. Cells expressing MVI siRNA had a density of approximately 2±0.3 spines per 10 µm in the dendrites (*n = *13 cells), and no spines were seen on the axons (*n = *13 cells; Figure S8). And in all cells expressing either dnMVI or MVI siRNA, the axon was clearly identifiable as being the longest process and being untapered except at the initial segment, in dramatic contrast to the dendrites (Figure S8). Note that in those experiments where the distribution of Ankyrin G was unpolarized, the above morphological distinctions were used to identify the axon and the dendrites, based on published methods [23]. Note also that in experiments where the localization of Ankyrin was disrupted by HA-dnMVI, the expression level of Ankyrin G (16±3 arbitrary units [a.u.], *n = *14) was not significantly diminished compared to the control condition (23±3 a.u., *n = *12, *p*>0.07). Thus, our results suggest that neuronal polarity is compatible with mislocalized Ankyrin G, but do not contradict previous experiments that show that polarity requires the presence of Ankyrin G [23].

### Myosin VI Mediates Axonal Enrichment through Dendrite-Specific Endocytosis

Several different studies have established that axonal proteins are initially transported to dendrites as well as axons, and then endocytosed preferentially from the dendritic surface [1],[3],[4]. Because Myosin VI is known to play a role in endocytosis, we tested whether it might mediate enrichment on the axonal membrane relative to the surface of the dendrites at least in part through this mechanism. As a first test of whether endocytosis is involved in the localization of Myosin VI we compared the distributions of surface versus intracellular CD8-MVIBD in transfected neurons. If Myosin VI mediates dendrite-specific endocytosis, we would expect that the intracellular distribution of CD8-MVIBD would be nonspecific, in contrast to the distribution of surface protein, which is concentrated to the axon. In fact this is the case: CD8-MVIBD on the cell surface was much more strongly localized to the axon (unnormalized ADR [uADR]  = 3.0±0.4, *n = *11) than was intracellular CD8-MVIBD (uADR  = 0.7±0.06; Figure S9). This result is consistent with CD8-MVIBD being concentrated at the axonal surface through dendrite-specific endocytosis. Note that because we labeled both surface and intracellular protein in the same cells, we were not able to label a nonspecifically localized protein and thus we used an unnormalized ADR (see Materials and Methods).

**Figure 6:**
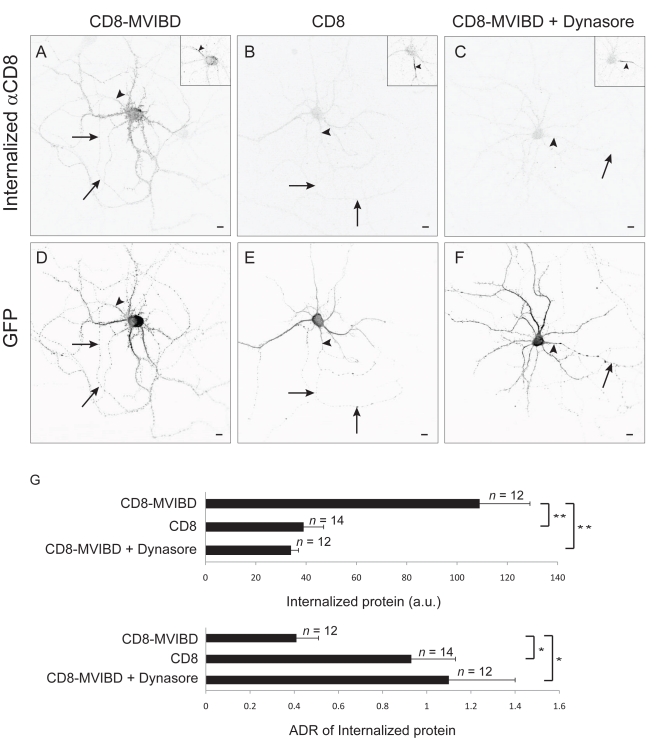
Interaction with Myosin VI promotes dendrite-specific endocytosis. (A) Internalized exogenous CD8-MVIBD that was labeled with anti-CD8 antibody and incubated for 30 min at 37°C in a cortical neuron in dissociated culture. Internalized protein was concentrated in the dendrites. (B) Internalized exogenous CD8 labeled under conditions identical to those in (A). (C) Internalized exogenous CD8-MVIBD labeled under conditions identical to those in (A), except with Dynasore, a blocker of endocytosis, included in the bath. (D–F) GFP labeling of the neurons in (A–C), respectively. Insets show staining of endogenous Ankyrin G. (G) Significantly more CD8-MVIBD than CD8 was internalized from cortical neurons in culture in a 30-min time frame. Internalization of CD8-MVIBD could be blocked by the presence of Dynasore in the medium. ADRs of CD8 versus CD8-MVIBD, which are significantly different, indicate that interaction with Myosin VI causes endocytosis to occur preferentially in the dendrites. ADRs of CD8-MVIBD in the presence versus absence of Dynasore were also significantly different. *, *p*<0.001; **, *p*<0.0003; Wilcoxon-Mann-Whitney test. Insets show staining of endogenous Ankyrin G. Arrows point to the axon; arrowheads point to the axon initial segment. Scale bars are 10 µm.

To more directly investigate whether Myosin VI contributes to the concentration of proteins at the axonal surface through dendrite-specific endocytosis, we tested whether interaction with Myosin VI affects the rate of internalization of surface protein. For these experiments, we measured the relative amount of CD8-MVIBD as compared with CD8 that is endocytosed from the surface of the axon or the dendrites during a 30-min time period. In live cortical neurons, exogenous CD8-MVIBD or CD8 was labeled with an antibody directed against an external epitope, and endocytosis of surface proteins was allowed to proceed for 30 min at 37°C. The neurons were then washed in acidic medium (pH = 2) in order to eliminate antibody that was bound to protein on the surface. Finally, the cells were fixed, and the internalized protein was stained under permeabilized conditions with a secondary antibody. To determine the effect of Myosin VI on endocytosis, we compared the amount of internalized CD8 versus CD8-MVIBD in each compartment. Overall, 2.5 times more CD8-MVIBD was internalized (109±20 a.u., *n = *12) as compared with CD8 alone (39±8 a.u., *n = *14, *p*<0.0003). Moreover, incubating the cells with Dynasore, a blocker of endocytosis, reduced the levels of endocytosed CD8-MVIBD to an amount similar to those obtained when CD8 was expressed alone (34±3 a.u., *n = *12, *p*<0.0001; Figures 6A–6G and S10) [25]. To determine whether interaction with Myosin VI produced an increase in dendritic as compared with axonal endocytosis, we compared the relative amount of protein that had internalized in the dendrites versus the axon. The ADR of internalized CD8-MVIBD under control conditions (0.41±0.11) was approximately 2.5-fold lower (more dendritic) than the ADR of CD8 (Figure 6G; 0.93±0.21) or of CD8-MVIBD in the presence of Dynasore (1.1±0.3), a significant difference (*p*<0.001) that indicates that interaction with Myosin VI causes dendrite-specific endocytosis. Note that this exposure to low pH did not appear to affect the localization of endogenous Ankyrin G or of Nav1.2, suggesting that it did not harm the cell (Figure S10).

**Figure 7:**
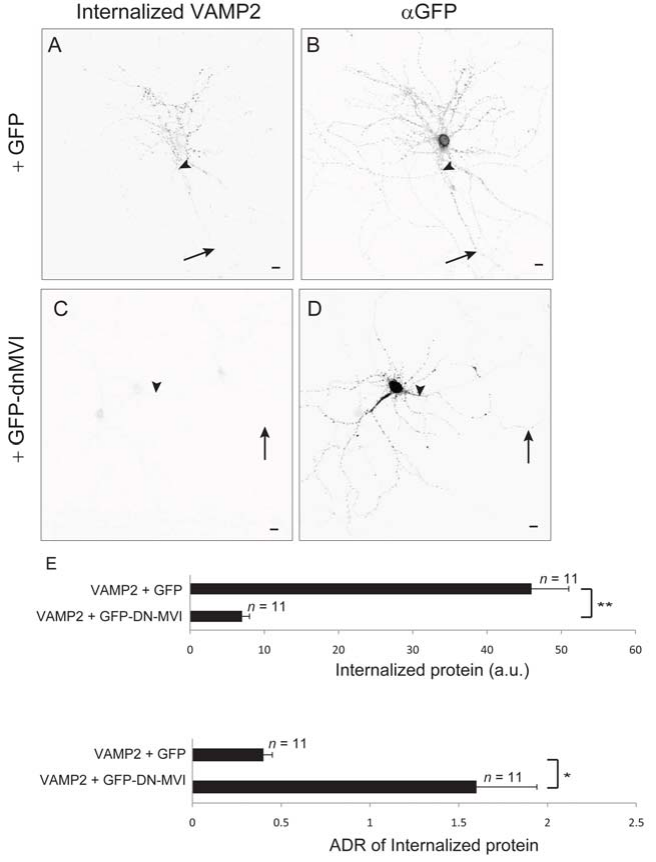
Interaction with Myosin VI is necessary for dendrite-specific endocytosis. Internalized exogenous VAMP2 (A) is localized primarily to dendrites in a cortical neuron coexpressing GFP (B). In contrast, much less VAMP2 (C) was internalized in cells coexpressing GFP-dnMVI (D). (E) Significantly more VAMP2 was internalized when it was coexpressed with GFP than when it was coexpressed with GFP-dnMVI. Furthermore, ADRs show that VAMP2 was internalized preferentially from the surface of the dendrites when coexpressed with GFP, but was internalized roughly equally from surfaces of the dendrites and from the axon when coexpressed with GFP-dnMVI. *, *p*<0.003; **, *p*<0.0001; Wilcoxon-Mann-Whitney test. Arrows point to the axon; arrowheads point to the axon initial segment. Scale bars are 10 µm.

**Figure 8:**
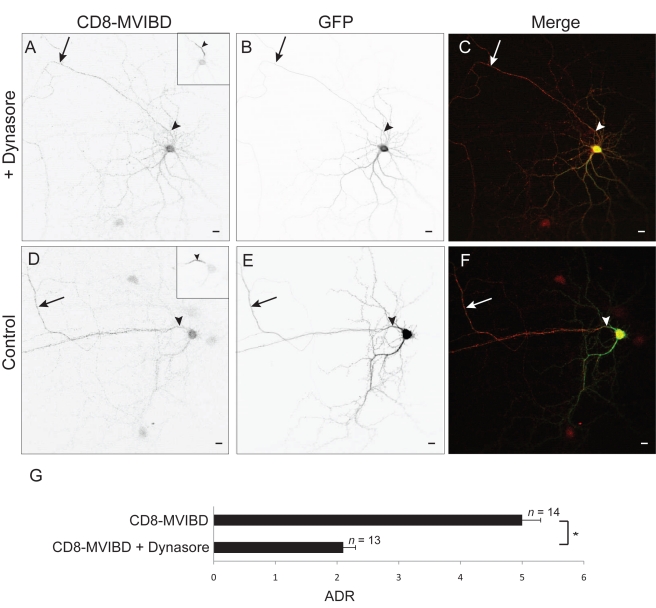
Axonal enrichment mediated by Myosin VI involves endocytosis. In a cortical neuron in dissociated culture in the presence of Dynasore, CD8-MVIBD (A) is expressed on the surface of both the axonal and somatodendritic compartments, although there is a preference for the axonal surface. (B) GFP coexpressed in the same cell as in (A). (C) Merge of CD8-MVIBD (red) and GFP (green). In contrast, CD8-MVIBD (D) is highly enriched on the surface of the axon when coexpressed with GFP (E) under control conditions. (F) Merge of CD8-MVIBD (red) and GFP (green). (G) The ADR of CD8-MVIBD is significantly reduced when it is expressed in the presence of Dynasore as compared with a control condition. *, *p*<0.0001. Insets show staining of endogenous Ankyrin G. Arrows point to the axon; arrowheads point to the axon initial segment. Scale bars are 10 µm.

Since it appears from the above experiments that interaction with Myosin VI promotes dendrite-specific endocytosis, we asked whether Myosin VI is necessary for endocytosis of exogenously expressed VAMP2, a protein that is enriched at the surface of the axon through a Myosin VI-dependent process (Figure 2). In experiments similar to those described in the previous paragraph, less VAMP2 was internalized when coexpressed with GFP-dnMVI (7±1 a. u., *n = *11; Figure 7A–7E) than when it was coexpressed with GFP (46±5 a.u., *n = *11), a significant difference (*p*<0.0001). Furthermore, the internalized protein was concentrated in the dendrites in the control condition (ADR  = 0.43±0.05), but was slightly concentrated in the axon when Myosin VI activity was blocked (Figure 7; ADR  = 1.6±0.3), a significant difference (*p*<0.003). This result indicates that Myosin VI is necessary for dendrite-specific endocytosis. Finally, we wanted to determine whether endocytosis is required for the concentration of proteins at the axonal surface that is mediated by Myosin VI. To this end, we tested the effect of the presence of the endocytosis blocker Dynasore on the surface distribution of CD8-MVIBD. When expressed in the presence of Dynasore, surface CD8-MVIBD was enriched on the surface of the axon (Figure 8A–8C; ADR  = 2.1±0.2, *n = *13), but to a much lesser extent than CD8-MVIBD under control conditions (Figure 8D–8G; ADR  = 5±0.3, *n = *14, *p*<0.0001). Together, our results suggest that interaction with Myosin VI increases the rate of endocytosis in the dendrites relative to the axon, which contributes to the concentration of protein on the axonal surface.

### Myosin VI Promotes Direct Trafficking to the Axon

In the previous experiments we noted that while blocking endocytosis reduced the degree to which the distribution of CD8-MVIBD was polarized, the protein still displayed enrichment in the axon (ADR  = 2.1±0.2, from above). Indeed, the ADR of surface CD8-MVIBD in the presence of Dynasore was still significantly larger than that of surface CD8 (ADR  = 0.80±0.13, *n = *12, *p*<0.001). Thus, eliminating endocytosis does not completely eliminate the relative enrichment of protein on the surface of the axon. We therefore wondered whether Myosin VI might play a role in direct trafficking of proteins to the axon. To explore this question, we looked specifically at whether interaction with Myosin VI caused proteins to travel preferentially to the axon during the initial stage of trafficking, before the protein is deposited on the plasma membrane. Accordingly, we generated cleavable tags on CD8 and CD8-MVIBD by attaching GFP to the extracellular N-terminal domains of each protein by means of a linker encoding a Thrombin cleavage site (tcs) (to give CD8-tcs-GFP and CD8-MVIBD-tcs-GFP). Following transfection of cortical neurons with these constructs, Thrombin was added to the medium so that the GFP tags were removed immediately upon arrival of the proteins on the cell surface. We confirmed that a substantial pool of protein, representing protein that had not yet reached the surface of the cell, could be visualized following fixation and permeabilization, while protein on the cell surface, visualized with surface staining without permeabilization, was nearly entirely cleaved (Figure S11). Thus, this method can be used to look at the initial stage of trafficking prior to delivery at the cell surface.

**Figure 9:**
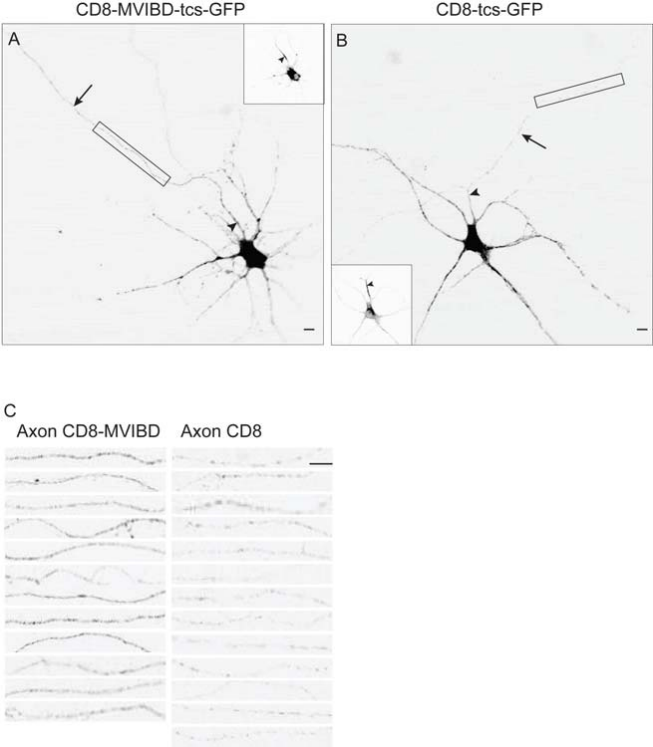
Interaction with Myosin VI promotes enrichment of intracellular protein in axon. (A) CD8-MVIBD-tcs-GFP, which is tagged on the extracellular N-terminus with a linker containing a Thrombin cleavage site, expressed in a cortical neuron with Thrombin in the medium and stained intracellularly with a rabbit anti-GFP antibody. The relative expression level of intracellular CD8-MVIBD-tcs-GFP in the axon is much higher than that of CD8-tcs-GFP (B), labeled under conditions identical to those in (A), indicating that interaction with Myosin VI promotes direct trafficking to the axon. (C) Comparison of axonal staining of CD8-tcs-GFP with CD8-MVIBD-tcs-GFP in axons. Top images in (C) correspond to areas surrounded by boxes in (A) and (B). Scale bars are 10 µm. Insets show staining of endogenous Ankyrin G. Arrows point to axon; arrowheads point to the axon initial segment.

Using this method we found internal CD8-MVIBD was over 3.5-fold more concentrated in the axon (uADR  = 0.62±0.07, *n = *12) than was comparable CD8 (uADR  = 0.17±0.02, *n = *13), a difference that is statistically significant (*p*<0.0001). Note that because we internally controlled for the success of the thrombin cleavage in every experiment, we could not also visualize the distribution of non-localized proteins and therefore calculated an unnormalized ADR (see Materials and Methods). Interestingly, in the case of both CD8 and CD8-MVIBD, the ADR was less than 1, indicating that protein is more concentrated in the somatodendritic region than in the axon. However, these figures must be interpreted with caution as the ER and Golgi are almost entirely located within the soma and dendrites (Figure S11; [26],[27]). Thus, the staining in the dendrites (Figure 9A and 9B) includes protein at the early stages of the secretory pathway prior to release from the Golgi as well as post-Golgi protein, whereas protein stained in the axon includes only post-Golgi protein. For this reason, the relative degree of axonal localization due to direct trafficking can be better appreciated by comparing the relative amounts of CD8 and CD8-MVIBD in the axon following exposure to Thrombin (Figure 9C). Interaction with Myosin VI causes enrichment of greater than 3-fold in the axon (axonal CD8-MVIBD  = 28±3 a.u., CD8 = 9±1 a.u.), a significant difference (*p*<0.0001), whereas the expression level of protein in the dendrites is comparable (dendritic CD8-MVIBD  = 94±10, CD8 = 81±11, *p*>0.3; Figure S11) with or without interaction with Myosin VI.

**Figure 10:**
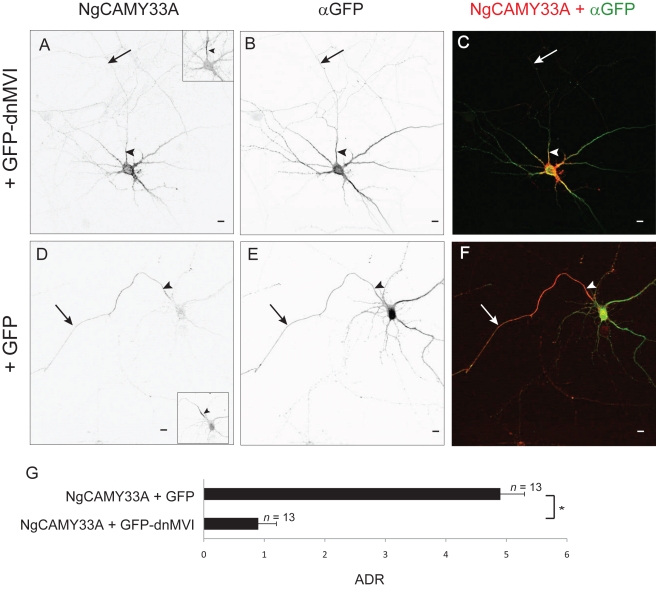
Myosin VI is necessary for endocytosis-independent localization of an axonal protein. In a cortical neuron in dissociated culture, surface NgCAMY33A (A) localizes nonspecifically when expressed with GFP-dnMVI (B). (C) Merge of NgCAMY33A (red) and GFP-dnMVI (green). In contrast, NgCAMY33A (D) localizes specifically to the axon when coexpressed with GFP (E). (F) Merge of NgCAMY33A (red) and GFP (green). (G) The ADR of NgCAM coexpressed with GFP is approximately 5-fold greater than when it is coexpressed with GFP-dnMVI. *, *p*<0.0001 (Wilcoxon-Mann-Whitney test). Insets show staining of endogenous Ankyrin G. Arrows point to the axon; arrowheads point to the axon initial segment. Scale bars are 10 µm.

To further test the hypothesis that Myosin VI contributes to direct axonal trafficking, we examined the effect of disrupting Myosin VI on a mutant of NgCAM (NgCAMY33A) that previously had been shown to be enriched on the surface of the axon independent of endocytosis [5]. NgCAMY33A localized specifically to axons when expressed in dissociated cortical neurons in combination with GFP (ADR  = 4.9±0.4, *n = *13) but was nonspecifically localized when expressed with GFP-dnMVI (Figure 10A–10G; ADR  = 0.9±0.3, *n = *13), results that are significantly different (*p*<0.0001). Thus, Myosin VI is necessary for the localization of an NgCAM mutant that traffics directly to the axon. Together, the experiments in Figures 9 and 10 provide evidence that Myosin VI contributes to the direct trafficking of transmembrane proteins to the axon.

### Interaction with Myosin VI Enriches Channelrhodopsin-2 on the Axonal Surface of Cortical Neurons In Vivo

**Figure 11:**
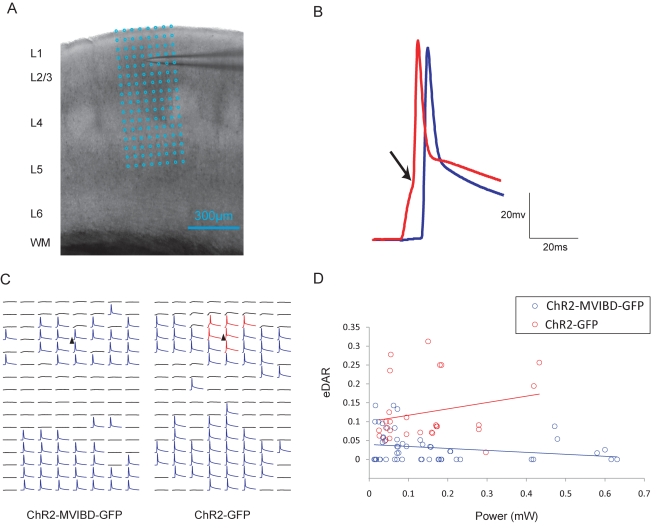
Interaction with Myosin VI promotes enrichment of ChR2 at the surface of axons of layer 2/3 pyramidal neurons. (A) Image of an acute brain slice with a recoding electrode. Cortical layers (L1 to L6) and white matter (WM) are indicated on the left. Blue grid represents 8×16 photostimulation patterns, with a spacing of 50 µm. (B) Schematic of waveforms triggered in soma and dendrites (red) and axons (blue). The arrow marks the clear inflection point (see Materials and Methods). (C) Example maps from a ChR2-MVIBD-GFP-positive cell (left) and a ChR2-GFP-positive cell (right). Black triangles mark soma locations. Blue traces indicate axonal excitation and red traces indicate somatic/dendritic excitation, respectively, using inflection point criteria. (D) The ratio of the number of APs evoked in soma and dendrites to the number of APs evoked in axons plotted against the laser power used for photostimulation. Blue circles represent the data points of the ChR2-MVIBD-GFP cells (*n = *6), and red circles represent the data points of the ChR2-GFP cells (*n = *8). The upward shift of the eDAR distribution of ChR2-MVIBD-GFP relative to that of ChR2-GFP is consistent with enrichment in axons of ChR2-MVIBD-GFP relative to ChR2-GFP.

In a previous study we showed that causing Channelrhodopsin-2 (ChR2), an ion channel that produces cationic currents in response to blue light [28], to interact with Myosin Va was sufficient to target it to the somatodendritic region of neurons in vivo, creating a new tool that can be used for circuit mapping [12]. Given that MVIBD can robustly enrich CD8-MVIBD at the surface of the axon (Figure 1), we asked whether it might similarly localize ChR2 in vivo. Using electrophysiological methods we compared the subcellular localizations of a fusion of ChR2, MVIBD, and GFP (ChR2-MVIBD-GFP) versus ChR2 (ChR2-GFP) in layer 2/3 pyramidal neurons of the somatosensory cortex. Experiments were performed on cortical slices cut from postnatal day 15–23 mice that were transfected at embryonic day 16 via in utero electroporation (Figure S12). By recording from layer 2/3 neurons intracellularly (and in the presence of CPP (5 μM) and NBQX (10 μM)) while stimulating with blue light in a raster pattern covering five cortical layers (Figure 11A), we mapped the locations where activation produced action potentials (APs) indicating the presence of a process of the recorded cell where ChR2 was present on the plasma membrane. Moreover, by examining the shape of the AP it was possible to determine whether the process that was stimulated was axonal or somatodendritic (see Materials and Methods; Figures 11B and S13) [12]. Note that all APs with characteristics indicating they were evoked at dendritic locations (*n = *175) were mapped to layers 2 and 3 and not to layers 4 and 5, which contain only axons of layer 2/3 cells, an indication that this classification method is accurate.

To gauge the relative localization of ChR2 constructs we compared the number of locations where APs were produced by stimulation of axons to the number produced by stimulation of dendrites at several different laser powers for each cell. Strikingly, two of six cells expressing ChR2-MVIBD-GFP showed excitation only in the axon (in comparison with zero of eight cells expressing ChR2-GFP; Figure 11C). In order to quantify relative localization of ChR2 versus ChR2-MVIBD, we calculated the ratio of the number of dendritic versus axonal excitation sites to give the electrophysiological dendrite to axon ratio (eDAR). Comparison of eDAR versus power for individual cells expressing ChR2-GFP versus ChR2-MVIBD-GFP (Figure 11D) shows that ChR2-MVIBD-GFP is present at a higher concentration in the axon than ChR2-GFP at virtually all laser powers tested. We assessed eDAR for each cell by averaging overall laser powers and found that ChR2-GFP localized with an eDAR over five times greater than that of ChR2-MVIBD-GFP (eDAR  = 0.13±0.03, 0.023±0.009; *n = *8, 6, respectively), a result that is statistically significant (*p*<0.001). Note that because the axons of layer 2/3 pyramidal neurons tend to be considerably larger than the dendrites [29], eDARs of ChR2-GFP tend to be smaller than 1 (Figure 11C). The eDAR results are consistent with maps of the expression patterns of ChR2-GFP and ChR2-MVIBD-GFP showing the presence of ChR2-MVIBD-GFP protein to a lesser extent in the dendrites and to an equal or greater extent in the axons when compared to ChR2-GFP (Figure 11C). Thus, fusion with the MVIBD is sufficient to cause ChR2 to be enriched on the surface of axons in vivo. Furthermore, ChR2-MVIBD allows neurons to be stimulated preferentially in the axon, a tool that will be useful for probing both the anatomical and functional properties of neuronal circuits.

## Discussion

The data in this study indicate that Myosin VI plays a role in the enrichment of transmembrane proteins at the axonal surface. Myosin VI facilitates the relative enrichment of proteins at the surface of the axon by increasing the rate of endocytosis within the somatodendritic compartment relative to the axonal compartment. Overall, our results are consistent with a model whereby axonal proteins are transported by kinesin motors to both the axon and the dendrites following release from the Golgi apparatus (Figure S14). Following arrival of protein at the somatodendritic plasma membrane, it is endocytosed through the actions of Myosin VI. This participation of Myosin VI in dendritic endocytosis of axonal proteins is consistent with numerous reports documenting its role in endocytosis in epithelial cells and neurons as well as in nonpolarized cells [15],[18],[30],[31]. It remains to be determined the exact mechanism whereby specificity of Myosin VI action is achieved. It is possible that Myosin VI interacts either directly or indirectly with axonal proteins, or, alternatively, it is possible that Myosin VI interacts with the endocytic machinery associated with vesicles carrying these proteins [32]. In the future it will be important to fully explore this question.

With the results of this study, there is now evidence that actin and myosin play pivotal roles in the concentration of proteins on the surface of either the axon or the dendrites; however, the role of microtubule-based mechanisms in these processes is less clear. A number of observations indicate that kinesin motors might be involved in steering vesicles to polarized compartments. For instance, the motor domain of Kif17, which transports dendritic proteins [6],[7], carries vesicles into the axon less efficiently than Kif5, which transports both axonal and dendritic proteins [33]. Tailless Kif5 targets to axons in a manner that is dependent on microtubule dynamics [11], and blocking Kif5 function with dominant negative constructs disrupts neuronal polarity [33]. Additionally, tyrosination, a phenomenon that occurs in dendrites, but not axons, causes Kif5 to bind at lower affinity to dendritic microtubules in comparison to those present in the axon [34],[35]. Finally, Kif5 prefers to bind to stable microtubules, which predominate in the axon, whereas other kinesins can also interact with unstable microtubules, which predominate in dendrites [36]. Although these experiments suggest that Kif5 has a bias towards axonal transport, it is also capable of carrying proteins such as GluR2 to the dendrites [8]. The work in this paper and our previous work would suggest that APP, which is also carried by Kif5 [37], and GluR2 localize differently because APP is carried in vesicles that associate with active Myosin VI, whereas vesicles carrying GluR2 likely are influenced by the actions of a plus-end-directed Myosin, such as Myosin Va [12]. Nonetheless, our experiments do not rule out an active role for kinesins in polarized targeting. For instance, it has been shown that Kif5 likely plays a role in the initial establishment of neuronal polarity during early development in vitro when it carries C-Jun N-Terminal Kinase to the nascent axon [38],[39]. Thus, under certain circumstances, kinesin motors are capable of targeting specifically to one or the other polarized compartment. However, the results in this paper and our previous study [12] would indicate that in many cases contributions by myosin motors are required for polarized trafficking.

In addition to kinesins, the dynein complex has also been suggested to participate in the polarized targeting of dendritic proteins, since causing organelles to link to dynein results in their transport specifically to dendrites [40]. While this effect is remarkably robust, it is not surprising given the fact that only dendrites contain microtubules that are oriented in a direction that permits movement of dynein away from the cell body and into the process [41]. Although blocking dynein with Dynamitin blocked dendritic targeting of GluR2, this disruption was not distinguished from the disruption of neuronal polarity in general. In contrast, blocking the function of Myosin Va or Myosin VI specifically disrupts the polarized distribution of subsets of proteins without destroying the overall polarity of the cell ([12]; Figures 2 and S6–S8). Furthermore, the assertion that dendritic proteins are specifically transported by dynein motors and not by kinesins would appear to contradict numerous studies [6]–[8],[42]. A model where dynein plays a crucial role in the maintenance of the cytoskeletal structure that is essential for neuronal polarity, while myosin motors in combination with kinesins mediate trafficking, is consistent with both our work and that of others.

Recent experiments support the existence of a vesicle filter dependent on actin within the axon initial segment. For instance, when large molecules are injected into the cell body of dissociated hippocampal neurons in culture, they are excluded from the axon in control neurons, but not in neurons exposed to Cytochalasin D, which promotes actin depolymerization [33]. Latrunculin B, which also promotes actin depolymerization, disrupts the localization of numerous polarized proteins including NgCAM [24]. Moreover, electron microscopy studies show the presence of actin filaments, albeit of indeterminate orientation, below the axolemma [43]. Our previous results suggested that this filter might play a role either in preventing entry of dendritic proteins into the axon [12],[44] or in promoting their entry into dendrites. In this study we found that a protein bound to Myosin VI (CD8-MVIBD) is localized intracellularly to both the axon and the dendrites (Figure S9), which would imply that there is nothing preventing it from entering the dendrites and thus that no vesicle filter exists in the proximal dendrites. In addition, a vesicle filter present in the axon initial segment that prevents proteins associated with Myosin Va from entering the axon would have the opposite effect on proteins associated with Myosin VI. Thus, Myosin VI might interact with actin filaments to guide vesicles containing axonal proteins and carried by kinesins towards the axon (Figure S14).

The presence of a vesicle filter in only one compartment could explain why axonal and dendritic proteins are trafficked by distinct mechanisms in mammalian neurons: (1) Axonal proteins associated with Myosin VI initially enter both compartments, but are concentrated in the axon in part through Myosin VI-dependent endocytosis from the dendritic plasma membrane. (2) Vesicles carrying dendritic proteins, which associate with Myosin Va, are prevented from entering the distal axon by its vesicle filter, which causes the vesicles to be trafficked directly to the dendrites. In invertebrates, however, it appears that localization of both axonal and dendritic proteins depends on endocytosis from the plasma membrane of the opposite compartment. Thus, in *Caenorhabditis elegans*, several dendritic receptors including the acetylcholine receptor and a glutamate receptor are localized to dendritic membranes through axon-specific endocytosis, while Synaptogyrin is localized to the axon through endocytosis from the somatodendritic membrane [45],[46]. It seems likely that in the relatively compact neurons of invertebrates, endocytic mechanisms alone are sufficient to localize transmembrane proteins efficiently to the membrane of either the axon or the dendrite. Thus, it is tempting to speculate that the advent in vertebrates of neurons with very long, thin axons dramatically increased the cost of localization strategies dependent on endocytosis. This development might have exerted sufficient selective pressure to facilitate the formation of a cytoskeletal structure, the vesicle filter, which prevents vesicles containing dendritic proteins from entering axons and promotes the entry of vesicles containing axonal proteins.

## Materials and Methods

### cDNA Constructs

VAMP2-YFP was constructed by inserting the sequence encoding amino acids 1–116 5′ to the gene encoding YFP. CD8-Nav was constructed by inserting the DNA sequence encoding amino acids 1839–1870 of NaV1.2 3′ to the sequence encoding amino acids 1–227 of CD8. GFP-dnMVI was constructed by inserting the DNA encoding the last 452 amino acids of rat Myosin VI 3′ to the GFP gene. HA-dnMVI was assembled by inserting the DNA encoding two hemagglutinin tags 5′ to the DNA encoding the final 452 amino acids of rat Myosin VI. The sequence for MVI siRNA is 5′-aacttcgaagtactggagccagcttcatc-3′ (OriGene). In Myosin VI, amino acids R660 and S661 are encoded by 5′-cgaagt-3′; in MVIr, R660 and S661 are encoded by 5′-cgctct-3′. CD8-Optineurin was constructed by placing the DNA encoding amino acids 1–227 of CD8 5′ to the DNA encoding Optineurin amino acids 420–526. CD8-DAB2 was constructed by placing the DNA encoding amino acids 1–227 of CD8 5′ to the DNA encoding DAB2 amino acids 649–719. CD8-MVIBD contains the DNA encoding amino acids 1–227 of CD8 5′ to the DNA encoding Optineurin amino acids 420–526 and the DNA encoding DAB2 amino acids 649–719. CD8-MVIBD-tcs-GFP and CD8-tcs-GFP were constructed by inserting the DNA encoding the GFP gene and the amino acids LVPRGS 5′ to the DNA encoding CD8-MVIBD and CD8, respectively. GFP-MVIBD was constructed by replacing the DNA encoding CD8 from CD8-MVIBD with the DNA encoding GFP. NgCAM (a generous gift from Gary Banker) was altered by placing a stop codon 5′ to the YFP gene. NgCAMY33A was constructed by mutagenizing the DNA encoding amino acid Y1186 to an alanine codon. GFP-GluR1and TfR-HA were previously described by Lewis et al [12]. Streptavidin (SA)–KDEL was made from GFP-KDEL [47], a gift from Katsuhiko Mikoshiba by substituting SA for GFP.

### Dissociated Cultures

Briefly, we removed the brains from day 18 embryos and dissected the cortices in Hanks balanced salt solution (Invitrogen) supplemented with 1 mM HEPES (Invitrogen). Cortices were dissociated in HBSS plus HEPES with 0.25% trypsin for 15 min and then washed three times for 5 min each with HBSS plus HEPES. The dissociated neurons were then plated on coverslips (22 mm ×22 mm, Fisher) at a density of 1×10^4^ or 5×10^4^ cells per well in neurobasal medium (Invitrogen) supplemented with 10 ml/l Glutamax (Invitrogen), 1 µg/ml gentamicin (Invitrogen), 20 ml/l B-27 supplement (Invitrogen), and 50 ml/l fetal bovine serum (Invitrogen). After 4 h the medium was replaced with serum-free neurobasal medium. All cells were transfected using CalPhos (Clontech) at 12–18 d in vitro using the manufacturer's suggested protocol. In order to determine the effect of dnMVI on exogenous proteins, it was expressed for 2 d, whereas its effect on endogenous proteins was determined after 7 d of expression. siRNA constructs were expressed for 14 d. Experimental protocols were conducted according to the United States National Institutes of Health guidelines for animal research and were approved by the Institutional Animal Care and Use Committee at the University of Southern California.

### Immunocytochemistry

The cells were fixed with 4% paraformaldehyde for 5 min and washed with PBS. This was followed by blocking with 1% bovine serum albumin, 5% normal goat serum, and 0.1% Triton X-100 in PBS. After blocking, primary antibody was diluted in blocking solution and added for 30–120 min. Secondary antibody was diluted in blocking solution and added for 30 min in the dark. Primary antibody concentrations were as follows: chicken anti-GFP (Aves), 1∶1,000; rabbit anti-GFP (BD Biosciences), 1∶1,000; mouse anti-HA (Covance), 1∶500; rabbit anti-Ankyrin G (Santa Cruz), 1∶1,000; mouse anti-Ankyrin G (Neuromab), 1∶500; mouse anti-CD8 (Dako), 1∶100; rabbit anti-Myosin VI (Proteus), 1∶100; rabbit anti-βIV Spectrin (a generous gift from Matthew Rasband), 1∶1,000; mouse anti-Nav1.2 (Neuromab), 1∶50; and mouse anti-Beta Tubulin (Sigma), 1∶1,000. The mouse anti-NgCAM (8D9, 1∶100), developed by Vance Lemmon, and the mouse anti-L1 (ASCS4, 1∶50), developed by Paul H. Patterson, were obtained from the Developmental Studies Hybridoma Bank initiated under the auspices of the National Institute of Child Health and Human Development and maintained by the Department of Biological Sciences, University of Iowa. Secondary antibodies were conjugated to Alexa 488, 594, or 647 fluorophores (Invitrogen).

### Surface Immunocytochemistry

Fresh neurobasal medium without serum and with primary antibody added was placed on the cells for 5–10 min. The cells were then treated as stated in the immunocytochemistry section. For the CD8-MVIBD intracellular versus surface immunocytochemistry, the coverslips were stained live for 5 min, then fixed and washed with PBS. Directly after the washes, Alexa 594 was added in block without Triton X-100 for 30 min. The cells were then blocked with Triton X-100 for 30 min, and stained for intracellular protein as stated in the immunocytochemistry section above.

### siRNA Experiments

Neurons were transfected as stated above with MVI siRNA, empty siRNA vectors, or MVI siRNA + MVIr. After 16 h, the cells were rinsed twice with NaCl HEPES buffer (140 mM NaCl, 5 mM KCl, 1 mM MgCl_2_, 24 mM D-glucose, 10 mM HEPES, and 1 mM CaCl_2_ [pH 7.4]) and placed into conditioned neurobasal medium for 13 d. On the 14th day, the cells were fixed and stained as stated in the immunocytochemistry section above.

### Internalized Anti-CD8 Experiments

Neurons were transfected as above with CD8-MVIBD or CD8. Forty minutes prior to addition of antibody either DMSO or 15 µM Dynasore (CalBioChem) was added to each well. After 48 h of expression, mouse anti-CD8 was added to the medium for 30 min at 37°C. Following antibody feeding, the cells were washed with pH 2 medium for 2 min. The cells were then stained as explained above. Final DMSO concentration in the medium was 0.1%.

### Endocytosis Block by dnMVI

Neurons were transfected as above with VAMP2-YFP and either GFP or GFP-dnMVI. After 48 h of expression, chicken anti-GFP was added to the medium for 30 min at 37°C. Following antibody feeding, the cells were washed with pH 2 medium for 2 min. The cells were then fixed and stained for intracellular protein with rabbit anti-GFP and anti-Ankyrin G.

### Thrombin Cleavage Experiments

Neurons were transfected as stated above with CD8-MVIBD-tcs-GFP or CD8-tcs-GFP. After 6 h, the cells were placed in conditioned medium equilibrated for at least 2 h to 10% CO_2_ at 37°C for 15 min in 5% CO_2_ at 37°C to remove excess CalPhos crystals. The neurons were then placed in conditioned medium containing 2 units/ml Thrombin (Sigma) for 16 h. Lastly, the neurons were surface stained, as previously stated, with chicken anti-GFP, then fixed and stained for intracellular protein with rabbit anti-GFP and anti-Ankyrin G.

### Biotinylated Thrombin

Thrombin (Sigma) was biotinylated with a biotinylation kit from Pierce. After transfection with DNA encoding GFP, biotinylated Thrombin was added to the cells at 2 units/ml Thrombin for 16 h. The cells were washed with 1× PBS, then fixed and stained with chicken anti-GFP and biotin-rhodamine (Invitrogen).

### Dynasore Experiments

Neurons were transfected as stated above with CD8-MVIBD-tcs-GFP and GFP. After 6 h, the cells were placed in conditioned medium equilibrated for at least 2 h to 10% CO_2_ at 37°C for 15 min in 5% CO_2_ at 37°C to remove excess CalPhos crystals. The neurons were then placed in conditioned medium containing either DMSO or 15 µM Dynasore (CalBioChem). After 24 h, the neurons were surface stained, as previously stated, with chicken anti-GFP, then fixed and stained for total protein with rabbit anti-GFP and anti-Ankyrin G. Final DMSO concentration in the medium was 0.1%.

### Myosin VI and GFP-MVIBD Co-Immunoprecipitation

COS cells were transfected via Effectene (Qiagen) with Myosin VI HA and either GFP-MVIBD or GFP alone. After 48 h, lysate was prepared in lysis buffer (150 mM NaCl, 1 mM EDTA, 10 mM Tris [pH 8.0], 1% NP40, 0.12 mg/ml PMSF, 2 µg/ml Leupeptin, 1 µg/ml Aprotinin, 10 mM NaF, and 1 µg/ml Pepstatin [all from Sigma]). The lysate was pre-cleared with agarose beads for 2 h at 4°C, and then 50 µl of goat anti-HA beads (Abcam) were added to 500 µg of lysate O/N at 4°C. The following day, the beads were washed five times with the lysis buffer above except the detergent was reduced to 0.1% NP40. The beads were collected in 5× SDS sample buffer and boiled for 5 min. Finally, the samples were then run on SDS-PAGE gels and transferred to nitrocellulose blot paper (Pall). The blots were then probed with either mouse anti-HA (Covance), 1∶3,000, or rabbit anti-GFP (BD Biosciences), 1∶2,000. Secondary antibodies used were goat anti-mouse HRP (Invitrogen), 1∶10,000, or goat anti-rabbit HRP (Invitrogen), 1∶5,000. The blots were developed with Western Lighting ECL-Plus (PerkinElmer) and transferred to film (Kodak).

### Myosin VI siRNA Biochemistry

COS cells were transfected via Effectene (Qiagen) with Myosin VI or MVIr and either empty siRNA or MVI siRNA. After 4 d, lysate was prepared as above. Finally, the samples were then run on SDS-PAGE gels and transferred to nitrocellulose blot paper (Pall). The blots were then probed with either mouse anti-HA (Covance), 1∶3,000, or mouse anti-β tubulin (Sigma), 1∶1,000. Secondary antibody used was goat anti-mouse HRP (Invitrogen), 1∶10,000. The blots were developed with Western Lighting ECL-Plus (PerkinElmer) and transferred to film (Kodak).

### Image Capture and Analysis

All imaging was done on a Bio-Rad MRC-1024 confocal microscope. Each cell was imaged as a single optical section. Each image was taken with a ×40 objective and at ×1 zoom, unless otherwise stated. None of the images used for quantification contained any saturated pixels in the axon or dendrites. Cells that had clearly definable axons and overall healthy morphology and were not obscured by neighboring cells were chosen on the basis of GFP or HA-mCherry staining. In general cells chosen for analysis had enriched Ankyrin G in the axon initial segment that allowed for identification of the axon; however there were three exceptions: (1) in the experiments where dendritic proteins were coexpressed with tagged dnMVI, where we purposely chose cells independent of Ankyrin G staining (Figure S7) to insure that we did not bias the results towards minimal effects; (2) in the experiments where NgCAM or CD8-MVIBD was coexpressed with MVI siRNA (Figures 4 and S4), because these experiments required a long incubation time, after which Ankyrin G localization was eliminated; and (3) in the experiments where the effect of dnMVI on endogenous proteins was assessed (Figures 3 and S5). These experiments required a long incubation time for endogenous proteins to turn over. In these cases we identified the axon as (1) the longest process, (2) lacking dendritic spines, and (3) untapered except for the proximal region. Note that similar criteria were used to identify the axon previously [23]. To test whether these criteria were sufficient to identify axons, 104 neurons were stained with a nonspecifically localized protein such as GFP or HA-mCherry and then counterstained with Ankyrin G. In each case, the axon was identified from the nonspecifically stained images. After comparison with the Ankyrin G staining, in each case it was confirmed that all 104 axons were successfully identified using the morphological criteria.

To quantify the degree of polarization in the distribution of a particular protein (P1) we calculated the ADR. We calculated two different forms of ADR, normalized (ADR) and unnormalized (uADR). In both cases, we calculated the mean amount of fluorescence per pixel in the entirety of the dendrites and in the axon distal to the initial segment (with background subtracted from both). We then calculated the ratio of these values to give the uADR, which for most nonspecifically localized proteins was somewhere between 0.2 and 0.3.
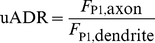
(1)where *F*_P1,axon_ is the average fluorescence intensity per pixel of protein P1 in the axon.

For normalized ADR, we then divided by the ratio of the mean fluorescence per pixel in the axon to the mean fluorescence per pixel in the dendrites of a nonspecifically localized protein such as GFP.
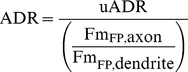
(2)where 
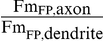
 is the mean fluorescence intensity per pixel associated with GFP or HA-mCherry in the axon or dendrite.

The advantage of calculating the normalized ADR is that it is simple to interpret, as ADR  = 1 indicates a protein is nonspecifically localized, ADR <1 indicates a dendritically localized protein, and ADR >1 indicates an axonally localized protein.

All measurements were done using ImageJ (US National Institutes of Health). All values of ADR were expressed ± the standard error of the mean. Comparisons of ADRs were made with the Wilcoxon-Mann-Whitney test, which can test the significance of nonparametric data. All analyses were performed blinded.

### In Utero Electroporation

Experimental protocols were conducted according to the US National Institutes of Health guidelines for animal research and were approved by the Institutional Animal Care and Use Committee at Janelia Farm Research Campus. Layer 2/3 pyramidal neurons in the barrel cortex were transfected via in utero electroporation. The plasmids for electroporation contained ChR2 fusion proteins (concentration, 2 µg/µl) and cytoplasmic mCherry at 5∶1 molar ratio.

### Slice Preparation

Postnatal day 15–23 mice were used in these experiments. Animals were deeply anesthetized with isofluorane. The brain was removed and placed in an ice-cold cutting solution containing 110 mM choline chloride, 25 mM NaHCO_3_, 25 mM D-glucose, 11.6 mM sodium ascorbate, 7 mM MgCl_2_, 3.1 mM sodium pyruvate, 2.5 mM KCl, 1.25 mM NaH_2_PO_4_, and 0.5 mM CaCl_2_. Coronal slices of the barrel cortex (300 µm thick) were cut with a vibrating slicer (Microm) and incubated in oxygenated ACSF (127 mM NaCl, 25 mM NaHCO_3_, 25 mM D-glucose, 2.5 mM KCl, 1 mM MgCl_2_, 2 mM CaCl_2_, and 1.25 mM NaH_2_PO_4_, aerated with 95% O_2_/5% CO_2_) for 45 min at 35°C before the recordings.

### Electrophysiology and Photostimulation

Recordings were performed at room temperature (22–24°C) in the presence of glutamate receptor blockers (CPP, 5 µM; NBQX, 10 µM). Electrophysiology and stimulus conditions were largely as described previously. The resting potentials of ChR2-MVIBD-GFP-positive cells and ChR2-GFP-positive cells were indistinguishable (ChR2-MVIBD-positive, −68±6 mV, *n = *6; ChR2, −66±6 mV, *n = *6). The light source for photostimulation was with a blue laser (473 nm; Crystal Laser), delivered through an air immersion objective (4×; 0.16 NA; UPlanApo, Olympus). Photostimulation was with a beam diameter of 6–10 µm (scattering in the tissue was not taken into account) on an 8×16 grid with 50-µm spacing, except that three ChR2 venus cells were recorded on an 8×16 grid with 75-µm spacing. In this case, we took only the same total area as a 50-µm spacing grid would cover. Photostimuli consisted of light pulses with 1-ms durations and powers in the range 5–1,000 µW at the specimen. Spikes were recorded in whole-cell current clamp mode.

### Inflection Point Analysis for ChR2-MVIDB and ChR2-Expressing Neurons

Antidromic APs triggered in axons and triggered in soma and dendrites could be distinguished by their waveforms. To determine whether an AP evoked by photostimulation was dendritic or axonal, we utilized the charging phase that is characteristic of dendritic APs. For dendritic APs, this charging phase should reach the AP threshold (set to be 15 mV in this analysis) at the inflection points. Otherwise, lack of charging phase would be categorized as axonal APs. In order to determine whether there is a charging phase and the position of the inflection point, we calculated the first derivative of an AP trace. The AP is identified as a derivative peak larger than 25 mV/ms; the charging phase is identified as a derivative peak before the AP and larger than 0.5 mV/ms; and the inflection point was found as the lowest derivative point between the charging peak and the AP peak.

## Supporting Information

Figure S1 **Myosin VI is localized diffusely in both the axonal and somatodendritic compartments.** Staining of endogenous Myosin VI (A) in a cortical neuron in dissociated culture expressing GFP (B). Arrow points to the axon, arrowhead points to the axon initial segment. Insets show staining of endogenous Ankyrin G. Glial cell transfected with siRNA against Myosin VI (arrows) (C) and GFP (D) shows dramatically reduced staining of endogenous Myosin VI compared with an untransfected cell. Untransfected cell stained for Myosin VI shows dark labeling of a perinuclear structure characteristic of the Golgi apparatus (arrowheads). Scale bars are 10 µm.
(PDF)

Figure S2 **Structure and function of the MVIBD.** CD8 fused with the MVIBD of DAB2 localizes somewhat to axons (A), but is still present in dendrites in a cortical neuron coexpressing GFP (B). Similarly, CD8 fused with the MVIBD of Optineurin localizes somewhat to axons (C), but is still present in dendrites in a cortical neuron coexpressing GFP (D). (E) Schematic of CD8-MVIBD showing the Myosin VI binding sites of Optineurin and DAB2, which are fused in series to the C-terminus of CD8. (F) In COS cells cotransfected with GFP-MVIBD and HA-Myosin VI, immunoprecipitation with an anti-HA antibody coimmunoprecipitates both HA-Myosin VI and GFP-MVIBD. In contrast, when cells are transfected with GFP and HA-Myosin VI, immunoprecipitation with an anti-HA antibody precipitates only HA-Myosin VI.
(PDF)

Figure S3 **Expression of MVI siRNA dramatically reduces expression of Myosin VI.** Cortical neuron in culture expressing NgCAM (A), GFP (B), and siRNA directed against Myosin VI (C) exhibits diminished expression of Myosin VI. Endogenous Myosin VI is expressed in cells in dissociated cortical cultures at roughly 22% of the level that it is in cells expressing the empty siRNA vector. The amount of fluorescence associated with staining of Myosin VI in cells expressing MVI siRNA (Fl_MVI + MVI siRNA_) was 6±1 a.u., whereas for cells expressing the empty siRNA vector (Fl_MVI + empty vector_), it was 26±5 a.u. (D). This difference is significant (*p* < 0.0002, Wilcoxon-Mann-Whitney test). (E) When HA-Myosin VI is coexpressed with siRNA in COS cells, a Western blot stained with HA shows that there is virtually no HA-Myosin VI present. In contrast, when HA-Myosin VI is coexpressed with an empty siRNA vector or when MVI siRNA is coexpressed with an HA-tagged variant of Myosin VI that is impervious to siRNA (MVIr), there is HA staining indicating the presence of Myosin VI. Staining for Beta Tubulin indicates that equal amounts of protein were loaded in each case. *, *p* < 0.0001 (Wilcoxon-Mann-Whitney test). Arrow points to the axon. Scale bars are 10 µm.
(PDF)

Figure S4 **MVI siRNA blocks enrichment of CD8-MVIBD at the axonal surface.** CD8-MVIBD (A) is highly enriched on the surface of the axon in a neuron cotransfected with an empty siRNA vector and with GFP (B). (C) Merge of CD8-MVIBD (red) and GFP (green) from (A) and (B). In contrast, when CD8-MVIBD (D) is coexpressed with MVI siRNA and GFP (E), it localizes in a nonspecific manner. (F) Merge of CD8-MVIBD (red) and GFP (green) from (D) and (E). (G) ADR of CD8-MVIBD coexpressed with siRNA empty vector is 4-fold greater than that of CD8-MVIBD coexpressed with MVI siRNA. Scale bars are10 µm. *, *p* < 0.0001 (Wilcoxon-Mann-Whitney test).
(PDF)

Figure S5 **Blocking Myosin VI function blocks localization of axonal proteins.** Surface staining of exogenous NgCAM, exogenous CD8-Nav, and endogenous L1 (A, E, I) showed that each localized specifically to the axon when coexpressed with GFP (B, F, J). In contrast, surface NgCAM, CD8-Nav, and L1 (C, G, K) localized nonspecifically when coexpressed with GFP-dnMVI (D, H, L). Insets show staining of endogenous Ankyrin G. Arrow points to the axon; arrowhead points to the axon initial segment. Scale bars are 10 µm.
(PDF)

Figure S6 **Ankyrin G ADR is uncorrelated with ADRs of axonal proteins coexpressed with dnMVI.** Scatter plots of ADRs of CD8-Nav (A), NgCAM (B), and VAMP2 (C) versus ADR of Ankyrin G for individual cortical neurons coexpressing GFP-dnMVI. Correlation coefficients (*R* ^2^ < 0.03) indicate that the ADRs of the axonal proteins and that of Ankyrin G are uncorrelated.
(PDF)

Figure S7 **Disruption of Myosin VI function or expression does not disrupt dendritic targeting.** GFP-GluR1 (A) localized to the dendrites when coexpressed with HA-dnMVI (B), as did GFP-GluR1 (C) coexpressed with HA-mCherry (D), TfR-HA (E) when coexpressed with GFP-dnMVI (F), surface GluR1 (G) when coexpressed with HA-dnMVI (H), and GFP-GluR1 (I) when coexpressed with MVI siRNA and HA-mCherry (J). (K) Comparison of ADRs indicates that blocking Myosin VI function or its expression with a dominant negative variant of Myosin VI does not significantly disrupt dendritic targeting. ns, *p* > 0.1. Arrow points to the axon. Scale bars are 10 µm.
(PDF)

Figure S8 **Neurons expressing MVI siRNA maintain polarized morphology.** (A) Cortical neuron expressing siRNA against Myosin VI for 14 d. (B) High-power image of cell in (A) showing that dendrites display a tapered morphology and the presence of spines. (C) High-power image of cell in (A) and (B) showing the axon with an untapered morphology and the absence of spines. Note that arrowhead points to an autaptic connection. Arrows point to axon. Scale bars are 10 µm.
(PDF)

Figure S9 **Intracellular and surface CD8-MVIBD localize differentially.** Intracellular CD8-MVIBD localizes nonspecifically (A), whereas surface CD8-MVIBD localizes specifically to the axon (B). (C) Merge of surface (red) and intracellular (green) CD8-MVIBD. Inset shows staining of endogenous Ankyrin G. Arrow points to the axon; arrowhead points to the axon initial segment. Scale bar is 10 µm. (D) The uADR (see Materials and Methods) of surface CD8-MVIBD is significantly different from that of the intracellular protein, which is higher than that of GFP. *, *p* < 0.0001 (Wilcoxon-Mann-Whitney test).
(PDF)

Figure S10 **Acid wash does not affect the localization of endogenous proteins.** (A) High-power image of internalized CD8-MVIBD taken from cell in Figure 6A. (B) Cortical neuron expressing GFP and exposed to pH 2 medium for 2 min in the same manner as the neurons in Figure 6. (C and D) In the same neuron as in (B) endogenous Ankyrin G and endogenous Nav1.2 showed appropriate staining in the axon initial segment, indicating that the low pH treatment had not disrupted their localizations. Scale bars are 10 µm.
(PDF)

Figure S11 **Interaction with Myosin VI promotes direct trafficking to the axon.** (A) CD8-MVIBD-tcs-GFP, which is tagged on the extracellular N-terminus with a linker containing a Thrombin cleavage site, expressed in a cortical neuron with Thrombin in the medium and stained intracellularly with a rabbit anti-GFP antibody. The relative expression level of intracellular CD8-MVIBD-tcs-GFP in the axon is much higher than that of CD8-tcs-GFP (B), labeled under conditions identical to those in (A), indicating that interaction with Myosin VI promotes direct trafficking to the axon. (C and D) Surface staining with anti-GFP (using a monoclonal antibody) shows that almost 100% of the surface protein was cleaved. (E) Comparison of dendritic regions taken from 12 cells expressing CD8-MVIBD and 13 cells expressing CD8, showing that expression in the dendrites is at comparable levels. Note that each panel corresponds to the same cells as the panel in the same position in Figure 9. (F) Cortical neuron coexpressing GFP and SA-KDEL, an ER marker, show that the ER is concentrated in the soma and dendrites and very sparse in the axon. Note that SA-KDEL was stained using biotin-rhodamine. Ankyrin staining is shown in the inset. Arrow points to axon; arrowhead points to axon initial segment. (G) Cortical neurons were exposed to biotinylated Thrombin in the bath under conditions identical to those for the experiments in (A–E) and in Figure 9. Subsequently, the cell was fixed, permeabilized, and stained using SA-rhodamine to determine whether the Thrombin had been internalized. The lack of staining in the right panel corresponding to GFP staining in the left panel indicates that very little if any Thrombin was internalized.
(PDF)

Figure S12 **ChR2-MVIBD expression in slices of cortex following in utero electroporation.** Brain section (50 µm thick) from a 4-wk-old mouse electroporated at embryonic day 16 with ChR2-MVIBD-GFP and mCherry, which labeled layer 2/3 (L2/3) neurons. These neurons had exuberant axons in layer 2/3 and layer 5, consistent with prominent ChR2-MVIBD-GFP staining in layer 2/3 and layer 5 positions, while mCherry staining was strongest in layer 2/3 in somata. Also note that both intracellular and surface protein was labeled, so the expression pattern shown is not necessarily the same as that of surface protein. Scale bar is 100 µm.
(PDF)

Figure S13 **Histograms of membrane potentials at inflection points.** Histograms for (A) ChR2-MVIBD-GFP cells (*n  = * 6) and (B) ChR2-GFP cells (*n  = * 8). For each neuron, responses from all laser power stimulations are plotted. Red dashed lines in all cells indicate the threshold (15 mV) for determining axonal versus somatic/dendritic responses. In ChR2-GFP cells there is a small bump in higher membrane potentials, indicating the dendritic component, while this bump almost disappeared in ChR2-MVIBD-GFP cells. In general, there is a trend towards lower membrane potentials in ChR2-MVIBD-GFP cells than in the control ChR2-GFP cells.
(PDF)

Figure S14 **Myosin VI-dependent mechanisms for localization of axonal proteins.** Proteins are loaded into vesicles on the surface of the Golgi apparatus and from there are transported either to the axon or to the dendrites by kinesin motors (purple) on microtubules (blue). After the protein sent to the dendrites is deposited on the dendritic surface, it is endocytosed through the actions of Myosin VI (orange, Box B). Proteins loaded into vesicles that proceed to the axon are carried by kinesin motors along microtubules. It is possible that Myosin VI might also guide these vesicles towards the axon by moving along actin filaments (pink) with their plus ends oriented towards the cell body (Box A). In this paper we provide direct evidence that dendrite-specific endocytosis is involved in localization of axonal proteins. Other experiments suggest that a direct mechanism such as the one shown in Box A might also contribute to axonal trafficking, although additional experiments will be required to fully define this mechanism. Note that, for simplicity, only a single microtubule was drawn in the dendrite although there are, in fact, microtubules pointing in both directions.
(PDF)

## Abbreviations

AP: action potential
a.u.: arbitrary units
ADR: axon to dendrite ratio
ChR2: Channelrhodopsin 2
DAB2: Disabled homolog 2
dnMVI: dominant negative Myosin VI
eDAR: electrophysiological dendrite to axon ratio
ER: endoplasmic reticulum
GFP: green fluorescent protein
MVIr: Myosin VI rescue
MVI siRNA: Myosin VI short interfering RNA
MVIBD: Myosin VI binding domain
SA: Streptavidin
siRNA: short interfering RNA
tcs: Thrombin cleavage site
uADR: unnormalized axon to dendrite ratio
