# Supplementary figures and images for: A Role for Myosin VI in the Localization of Axonal Proteins
Source: PLoS Biol. 2011 Mar 1;9(3):e1001021. doi: 10.1371/journal.pbio.1001021 (PMC3046960; doi:10.1371/journal.pbio.1001021)

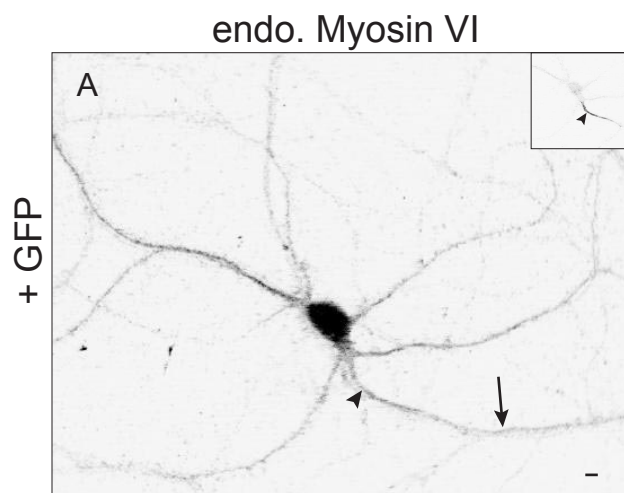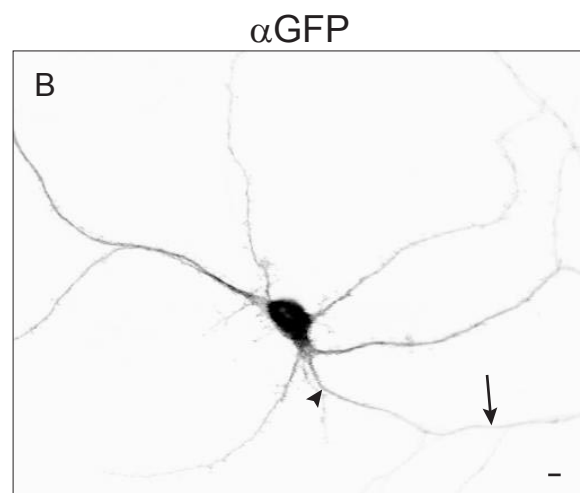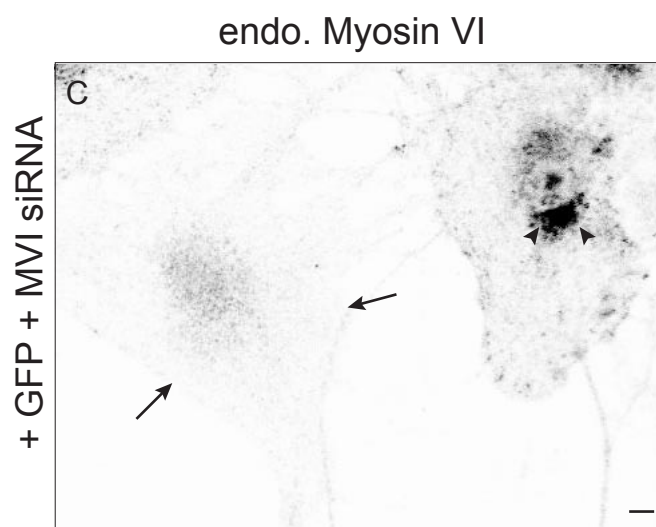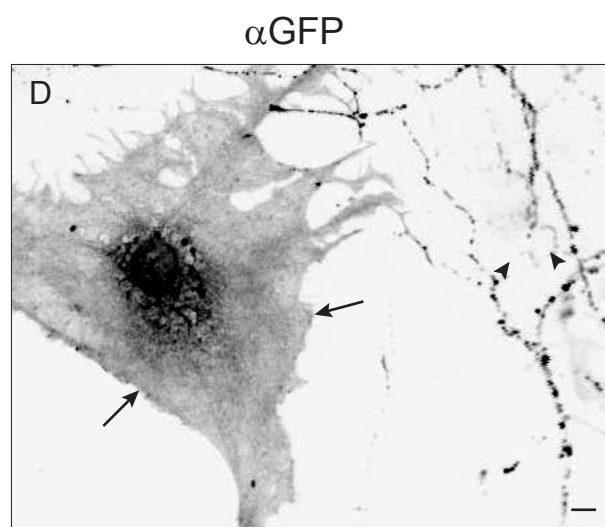

Supplement: Figure S1 — Myosin VI is localized diffusely in both the axonal and somatodendritic compartments. Staining of endogenous Myosin VI (A) in a cortical neuron in dissociated culture expressing GFP (B). Arrow points to the axon, arrowhead points to the axon initial segment. Insets show staining of endogenous Ankyrin G. Glial cell transfected with siRNA against Myosin VI (arrows) (C) and GFP (D) shows dramatically reduced staining of endogenous Myosin VI compared with an untransfected cell. Untransfected cell stained for Myosin VI shows dark labeling of a perinuclear structure characteristic of the Golgi apparatus (arrowheads). Scale bars are 10 µm. (PDF) [file pbio.1001021.s001.pdf]

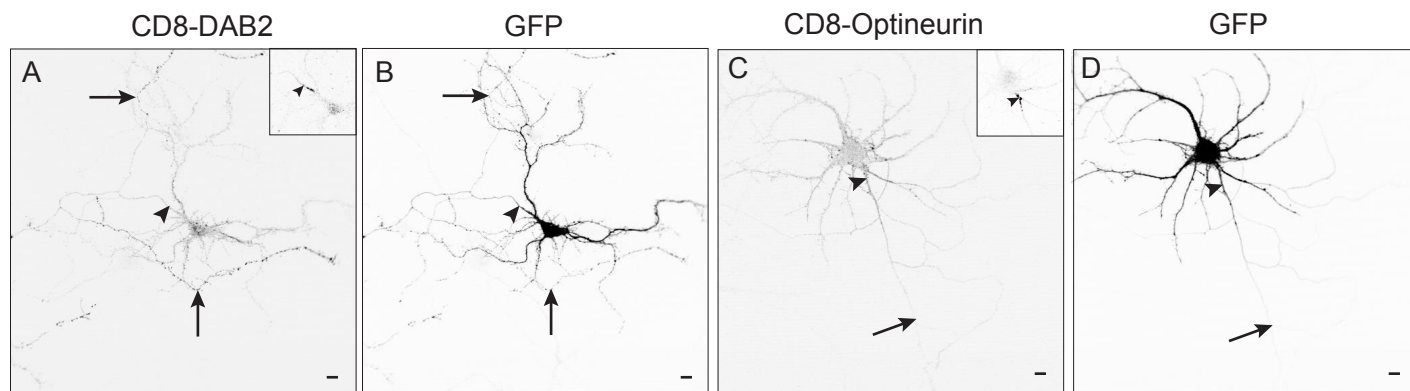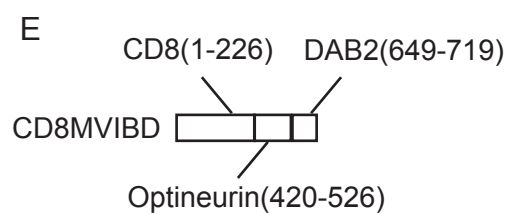

**F** Co-IP of GFP-MVIBD and GFP with HA-MyosinVI

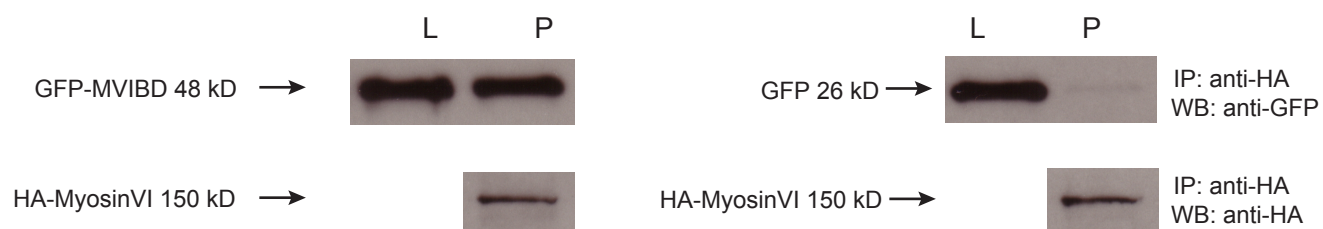

Supplement: Figure S2 — Structure and function of the MVIBD. CD8 fused with the MVIBD of DAB2 localizes somewhat to axons (A), but is still present in dendrites in a cortical neuron coexpressing GFP (B). Similarly, CD8 fused with the MVIBD of Optineurin localizes somewhat to axons (C), but is still present in dendrites in a cortical neuron coexpressing GFP (D). (E) Schematic of CD8-MVIBD showing the Myosin VI binding sites of Optineurin and DAB2, which are fused in series to the C-terminus of CD8. (F) In COS cells cotransfected with GFP-MVIBD and HA-Myosin VI, immunoprecipitation with an anti-HA antibody coimmunoprecipitates both HA-Myosin VI and GFP-MVIBD. In contrast, when cells are transfected with GFP and HA-Myosin VI, immunoprecipitation with an anti-HA antibody precipitates only HA-Myosin VI. (PDF) [file pbio.1001021.s002.pdf]

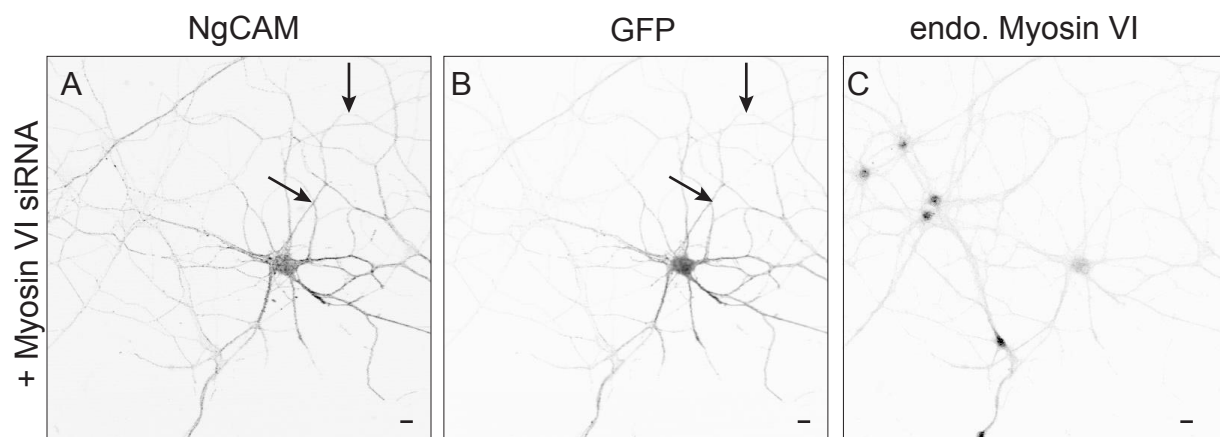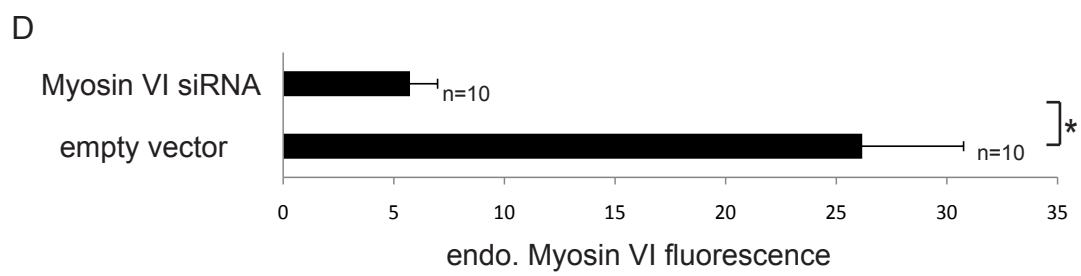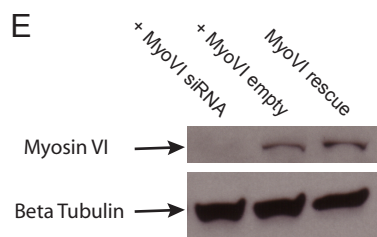

Supplement: Figure S3 — Expression of MVI siRNA dramatically reduces expression of Myosin VI. Cortical neuron in culture expressing NgCAM (A), GFP (B), and siRNA directed against Myosin VI (C) exhibits diminished expression of Myosin VI. Endogenous Myosin VI is expressed in cells in dissociated cortical cultures at roughly 22% of the level that it is in cells expressing the empty siRNA vector. The amount of fluorescence associated with staining of Myosin VI in cells expressing MVI siRNA (FlMVI + MVI siRNA) was 6±1 a.u., whereas for cells expressing the empty siRNA vector (FlMVI + empty vector), it was 26±5 a.u. (D). This difference is significant (p < 0.0002, Wilcoxon-Mann-Whitney test). (E) When HA-Myosin VI is coexpressed with siRNA in COS cells, a Western blot stained with HA shows that there is virtually no HA-Myosin VI present. In contrast, when HA-Myosin VI is coexpressed with an empty siRNA vector or when MVI siRNA is coexpressed with an HA-tagged variant of Myosin VI that is impervious to siRNA (MVIr), there is HA staining indicating the presence of Myosin VI. Staining for Beta Tubulin indicates that equal amounts of protein were loaded in each case. *, p < 0.0001 (Wilcoxon-Mann-Whitney test). Arrow points to the axon. Scale bars are 10 µm. (PDF) [file pbio.1001021.s003.pdf]

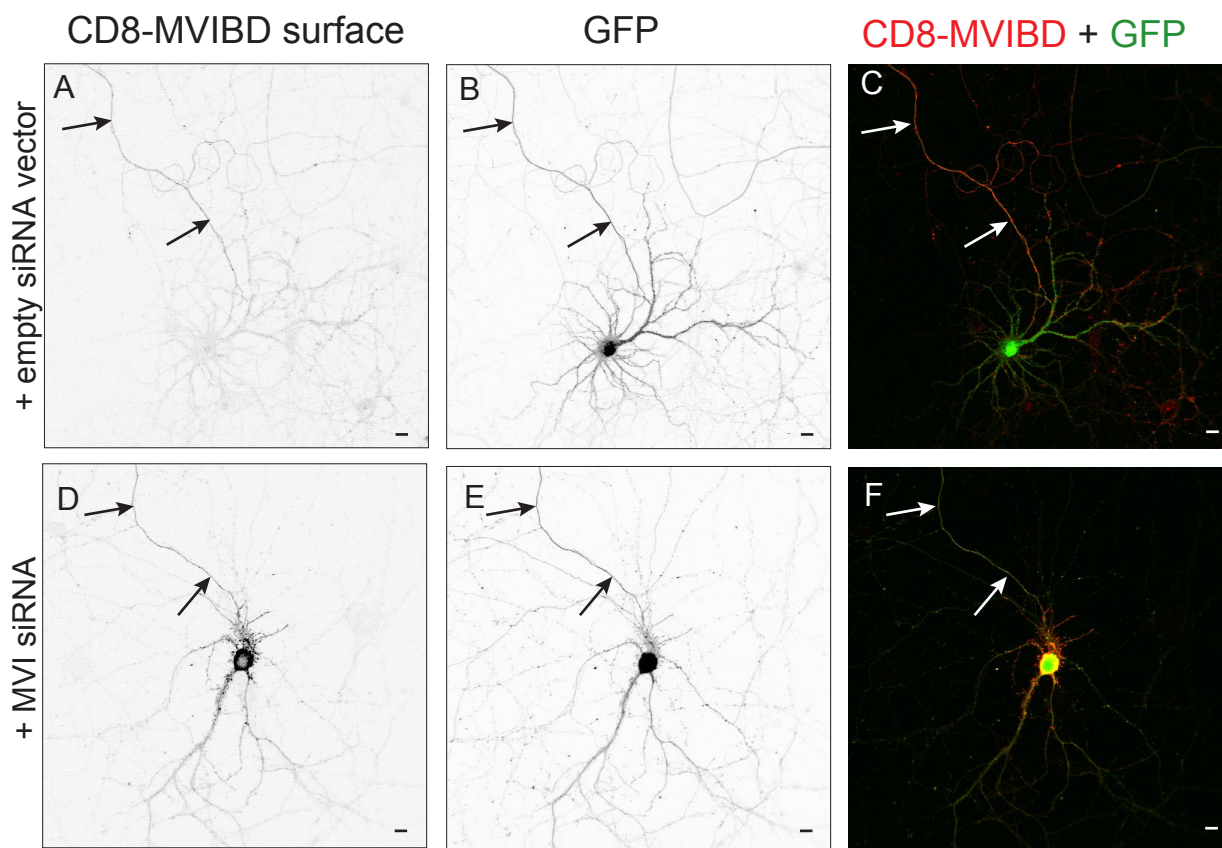

G

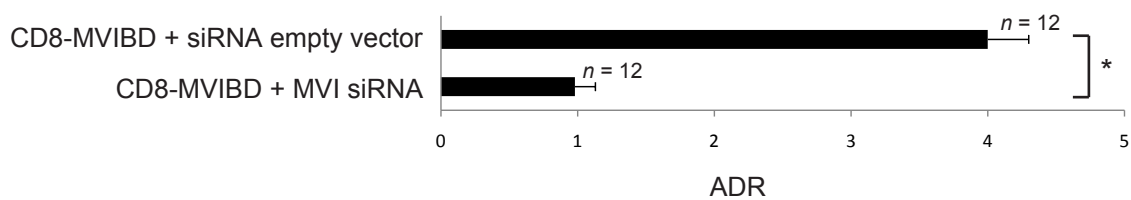

Supplement: Figure S4 — MVI siRNA blocks enrichment of CD8-MVIBD at the axonal surface. CD8-MVIBD (A) is highly enriched on the surface of the axon in a neuron cotransfected with an empty siRNA vector and with GFP (B). (C) Merge of CD8-MVIBD (red) and GFP (green) from (A) and (B). In contrast, when CD8-MVIBD (D) is coexpressed with MVI siRNA and GFP (E), it localizes in a nonspecific manner. (F) Merge of CD8-MVIBD (red) and GFP (green) from (D) and (E). (G) ADR of CD8-MVIBD coexpressed with siRNA empty vector is 4-fold greater than that of CD8-MVIBD coexpressed with MVI siRNA. Scale bars are10 µm. *, p < 0.0001 (Wilcoxon-Mann-Whitney test). (PDF) [file pbio.1001021.s004.pdf]

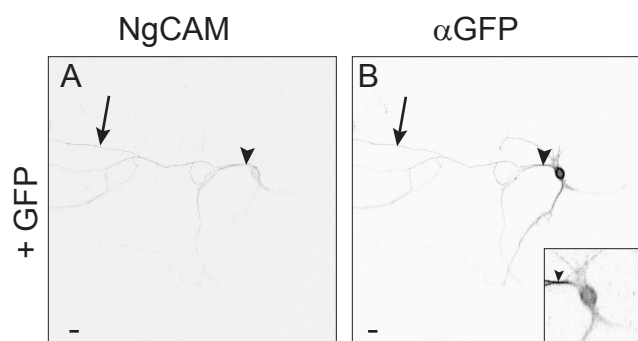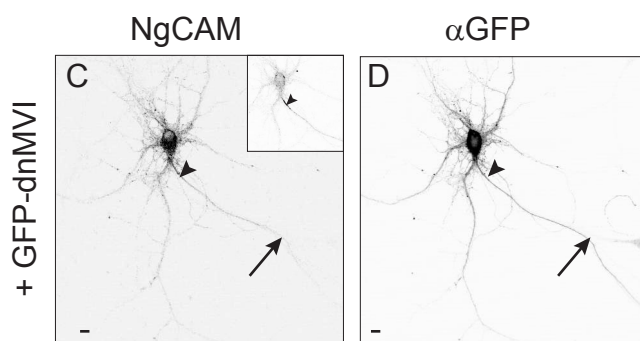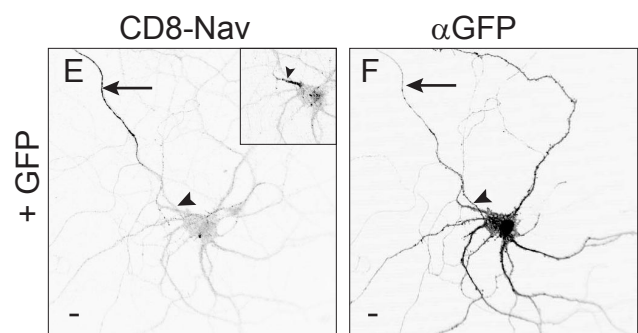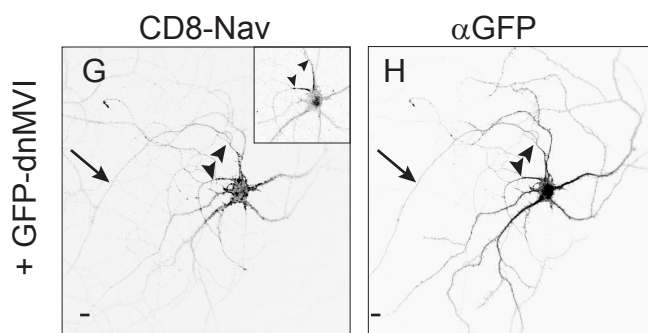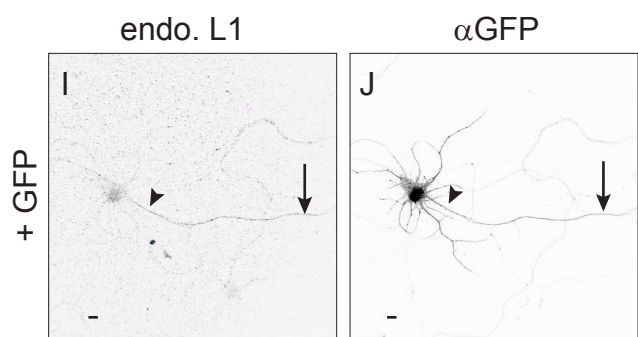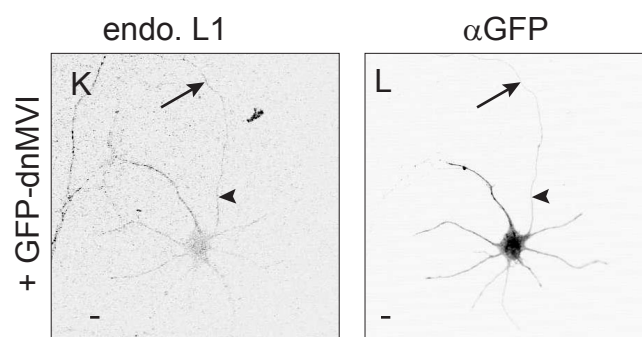

Supplement: Figure S5 — Blocking Myosin VI function blocks localization of axonal proteins. Surface staining of exogenous NgCAM, exogenous CD8-Nav, and endogenous L1 (A, E, I) showed that each localized specifically to the axon when coexpressed with GFP (B, F, J). In contrast, surface NgCAM, CD8-Nav, and L1 (C, G, K) localized nonspecifically when coexpressed with GFP-dnMVI (D, H, L). Insets show staining of endogenous Ankyrin G. Arrow points to the axon; arrowhead points to the axon initial segment. Scale bars are 10 µm. (PDF) [file pbio.1001021.s005.pdf]

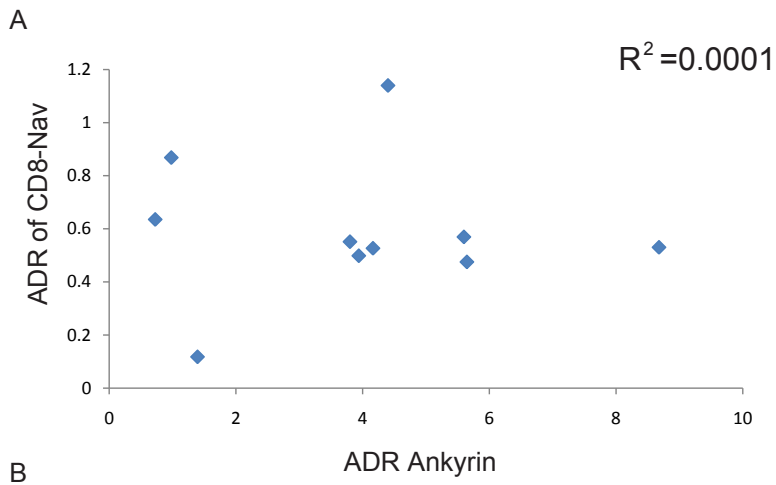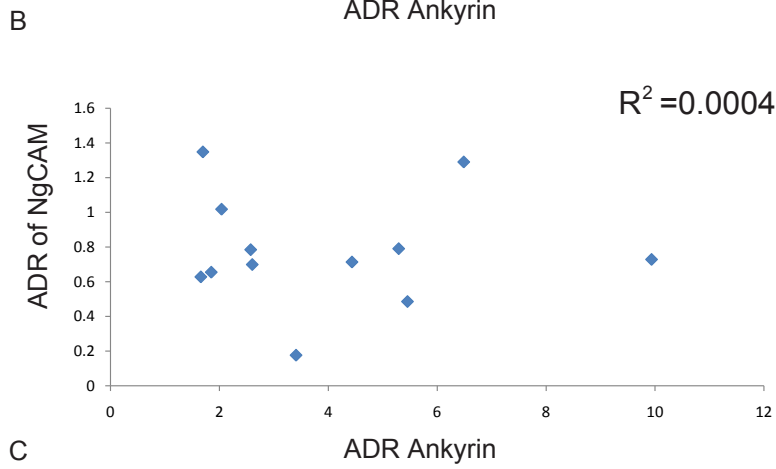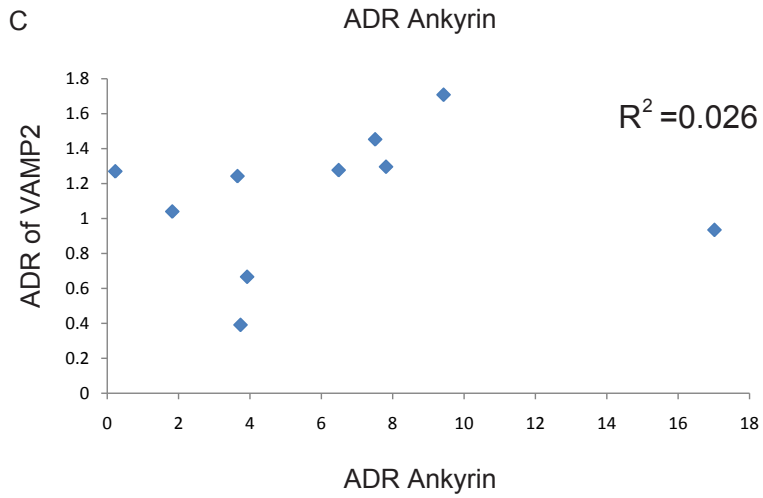

Supplement: Figure S6 — Ankyrin G ADR is uncorrelated with ADRs of axonal proteins coexpressed with dnMVI. Scatter plots of ADRs of CD8-Nav (A), NgCAM (B), and VAMP2 (C) versus ADR of Ankyrin G for individual cortical neurons coexpressing GFP-dnMVI. Correlation coefficients (R 2 < 0.03) indicate that the ADRs of the axonal proteins and that of Ankyrin G are uncorrelated. (PDF) [file pbio.1001021.s006.pdf]

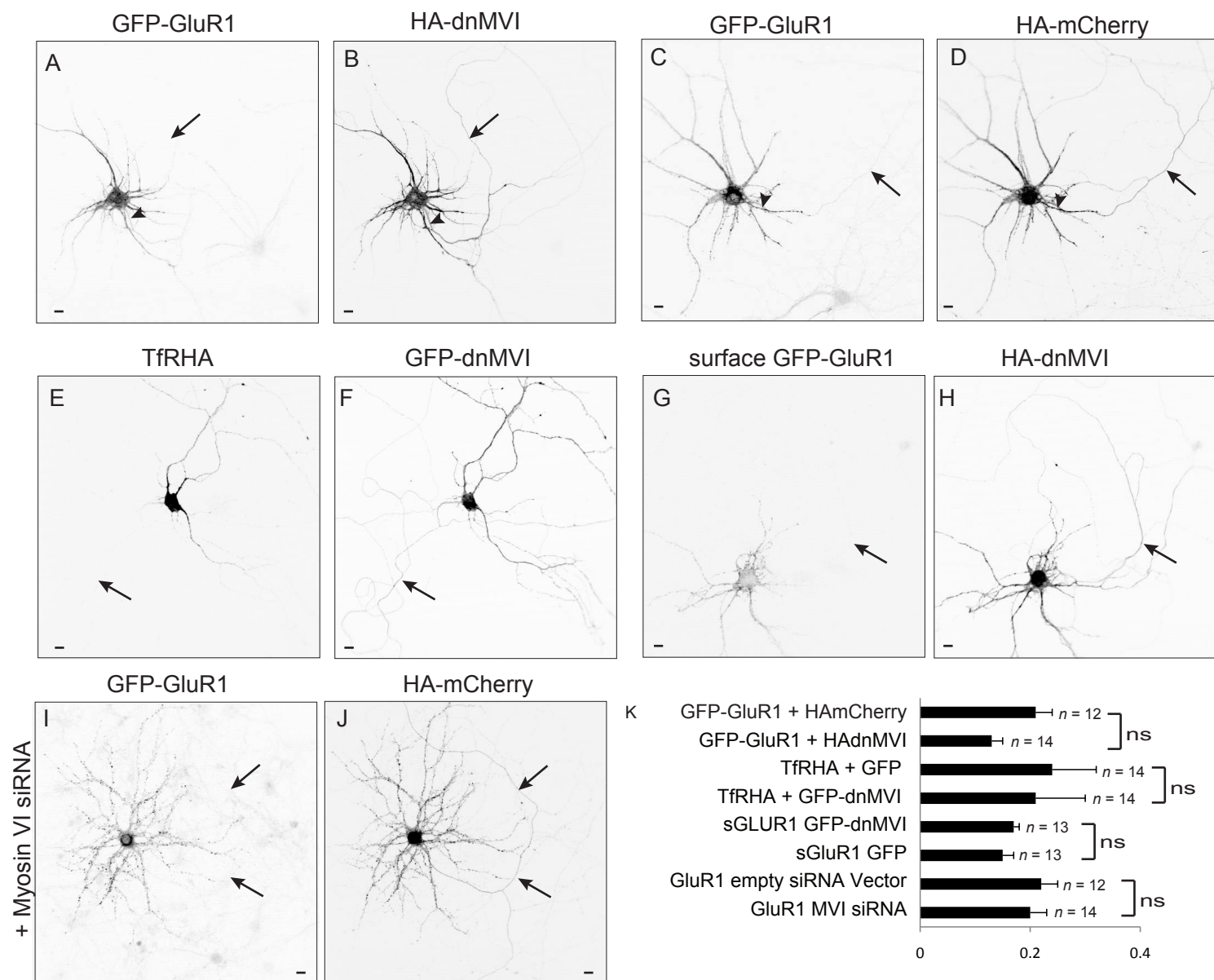

Supplement: Figure S7 — Disruption of Myosin VI function or expression does not disrupt dendritic targeting. GFP-GluR1 (A) localized to the dendrites when coexpressed with HA-dnMVI (B), as did GFP-GluR1 (C) coexpressed with HA-mCherry (D), TfR-HA (E) when coexpressed with GFP-dnMVI (F), surface GluR1 (G) when coexpressed with HA-dnMVI (H), and GFP-GluR1 (I) when coexpressed with MVI siRNA and HA-mCherry (J). (K) Comparison of ADRs indicates that blocking Myosin VI function or its expression with a dominant negative variant of Myosin VI does not significantly disrupt dendritic targeting. ns, p > 0.1. Arrow points to the axon. Scale bars are 10 µm. (PDF) [file pbio.1001021.s007.pdf]

Figure S9. Intracellular and surface CD8-MVIBD localize differentially.

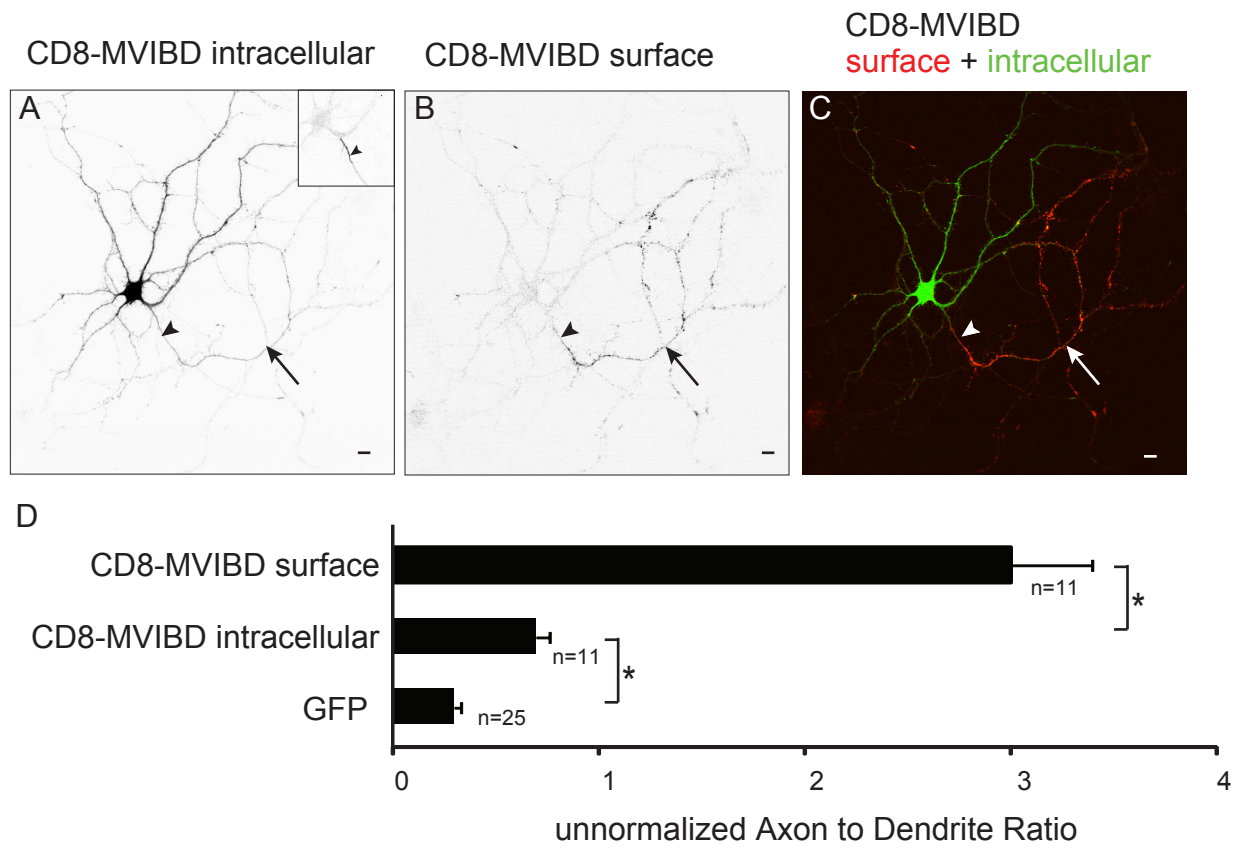

Supplement: Figure S9 — Intracellular and surface CD8-MVIBD localize differentially. Intracellular CD8-MVIBD localizes nonspecifically (A), whereas surface CD8-MVIBD localizes specifically to the axon (B). (C) Merge of surface (red) and intracellular (green) CD8-MVIBD. Inset shows staining of endogenous Ankyrin G. Arrow points to the axon; arrowhead points to the axon initial segment. Scale bar is 10 µm. (D) The uADR (see Materials and Methods) of surface CD8-MVIBD is significantly different from that of the intracellular protein, which is higher than that of GFP. *, p < 0.0001 (Wilcoxon-Mann-Whitney test). (PDF) [file pbio.1001021.s009.pdf]

Figure S10. Acid wash does not affect the localization of endogenous proteins.

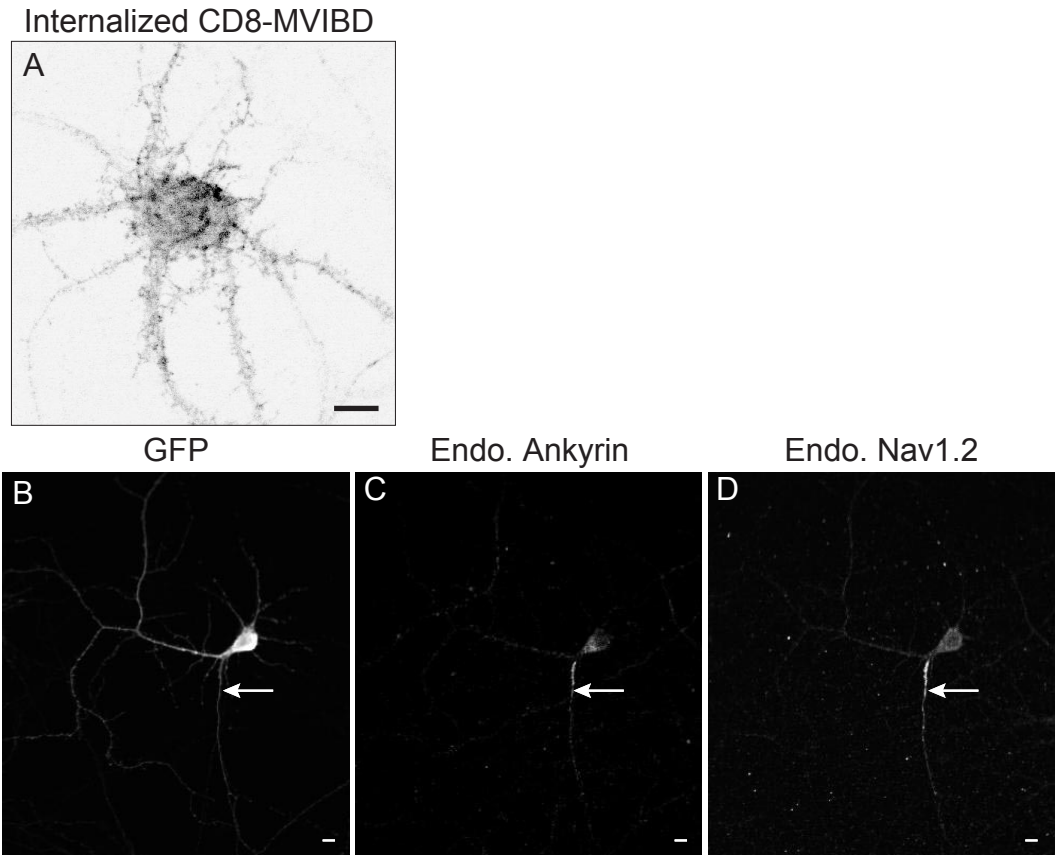

Supplement: Figure S10 — Acid wash does not affect the localization of endogenous proteins. (A) High-power image of internalized CD8-MVIBD taken from cell in Figure 6A. (B) Cortical neuron expressing GFP and exposed to pH 2 medium for 2 min in the same manner as the neurons in Figure 6. (C and D) In the same neuron as in (B) endogenous Ankyrin G and endogenous Nav1.2 showed appropriate staining in the axon initial segment, indicating that the low pH treatment had not disrupted their localizations. Scale bars are 10 µm. (PDF) [file pbio.1001021.s010.pdf]

Figure S11 Interaction with Myosin VI promotes direct trafficking to the axon.

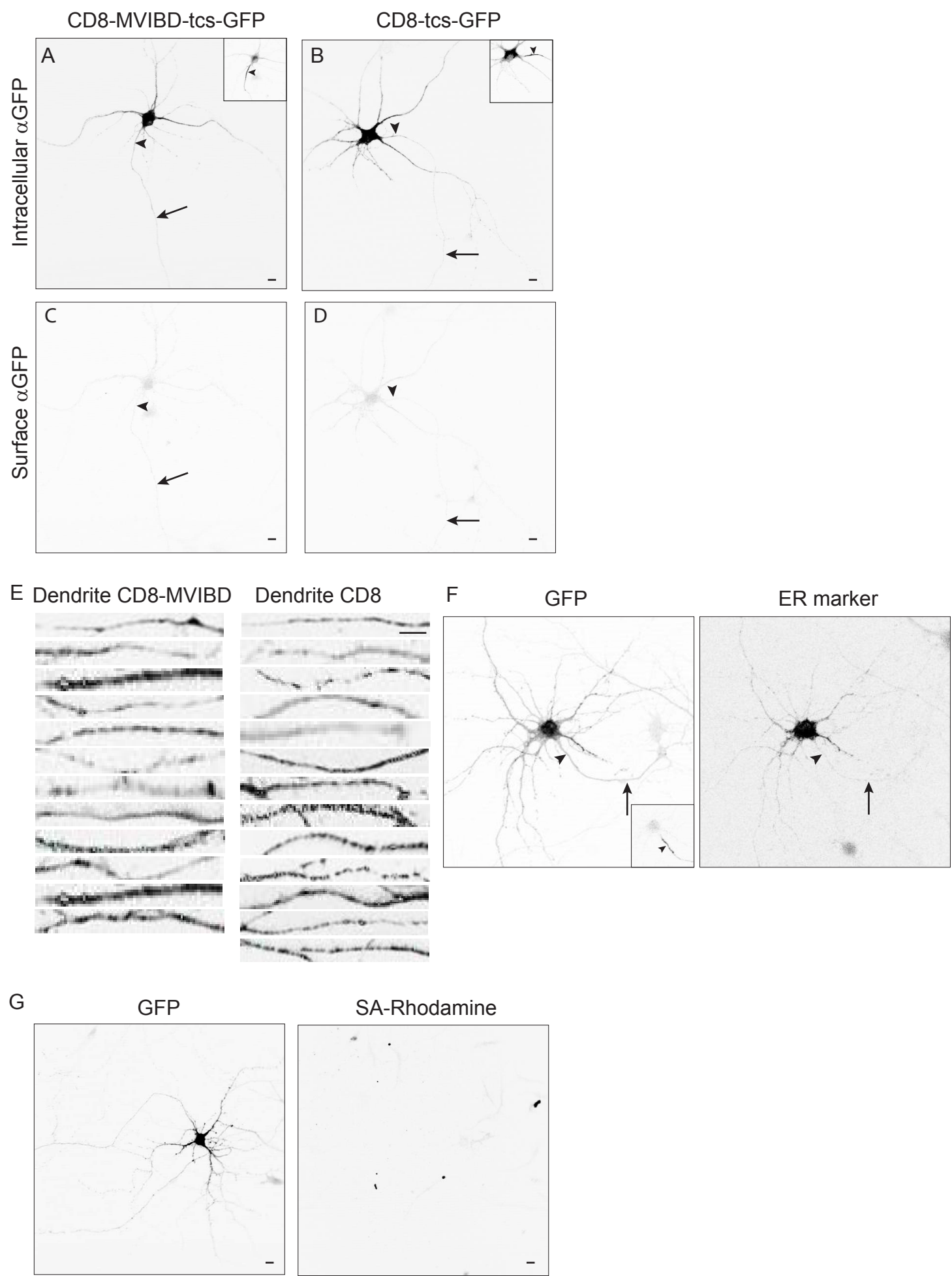

Supplement: Figure S11 — Interaction with Myosin VI promotes direct trafficking to the axon. (A) CD8-MVIBD-tcs-GFP, which is tagged on the extracellular N-terminus with a linker containing a Thrombin cleavage site, expressed in a cortical neuron with Thrombin in the medium and stained intracellularly with a rabbit anti-GFP antibody. The relative expression level of intracellular CD8-MVIBD-tcs-GFP in the axon is much higher than that of CD8-tcs-GFP (B), labeled under conditions identical to those in (A), indicating that interaction with Myosin VI promotes direct trafficking to the axon. (C and D) Surface staining with anti-GFP (using a monoclonal antibody) shows that almost 100% of the surface protein was cleaved. (E) Comparison of dendritic regions taken from 12 cells expressing CD8-MVIBD and 13 cells expressing CD8, showing that expression in the dendrites is at comparable levels. Note that each panel corresponds to the same cells as the panel in the same position in Figure 9. (F) Cortical neuron coexpressing GFP and SA-KDEL, an ER marker, show that the ER is concentrated in the soma and dendrites and very sparse in the axon. Note that SA-KDEL was stained using biotin-rhodamine. Ankyrin staining is shown in the inset. Arrow points to axon; arrowhead points to axon initial segment. (G) Cortical neurons were exposed to biotinylated Thrombin in the bath under conditions identical to those for the experiments in (A–E) and in Figure 9. Subsequently, the cell was fixed, permeabilized, and stained using SA-rhodamine to determine whether the Thrombin had been internalized. The lack of staining in the right panel corresponding to GFP staining in the left panel indicates that very little if any Thrombin was internalized. (PDF) [file pbio.1001021.s011.pdf]

Figure S12 ChR2-MVIBD expression in slices of cortex following in utero electroporation

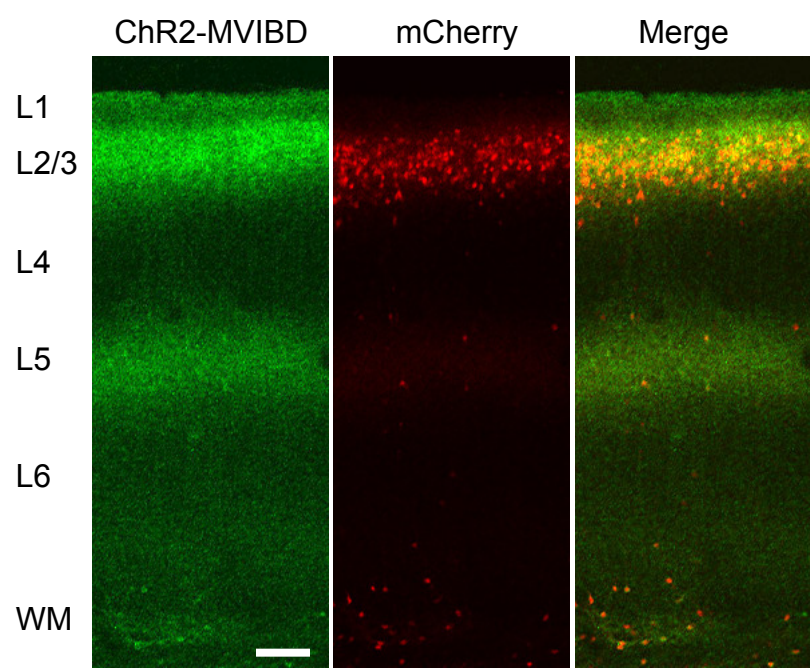

Supplement: Figure S12 — ChR2-MVIBD expression in slices of cortex following in utero electroporation. Brain section (50 µm thick) from a 4-wk-old mouse electroporated at embryonic day 16 with ChR2-MVIBD-GFP and mCherry, which labeled layer 2/3 (L2/3) neurons. These neurons had exuberant axons in layer 2/3 and layer 5, consistent with prominent ChR2-MVIBD-GFP staining in layer 2/3 and layer 5 positions, while mCherry staining was strongest in layer 2/3 in somata. Also note that both intracellular and surface protein was labeled, so the expression pattern shown is not necessarily the same as that of surface protein. Scale bar is 100 µm. (PDF) [file pbio.1001021.s012.pdf]

Figure S13. Histograms of membrane potentials at inflection points.

ChR2-MVIBD-GFP

A

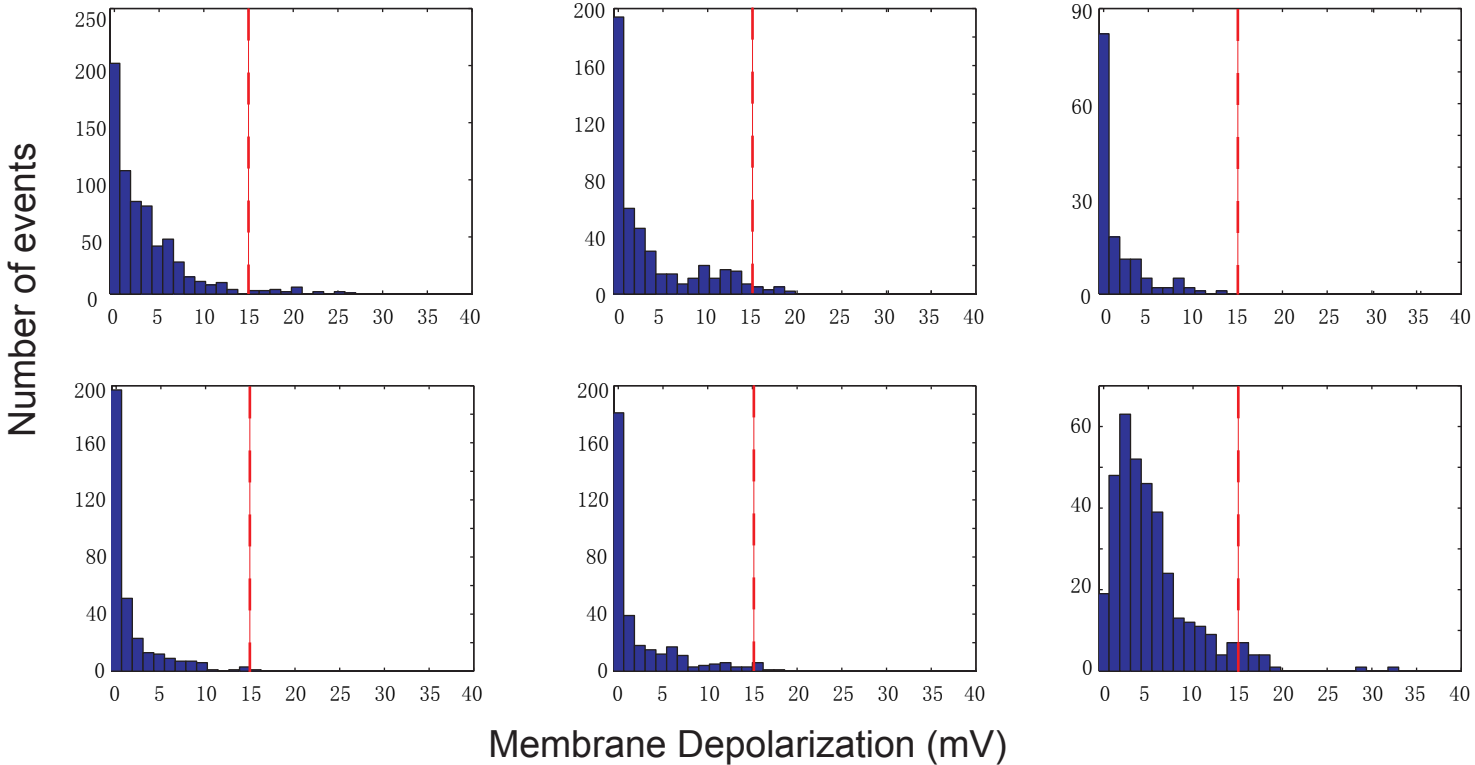

ChR2-GFP

B

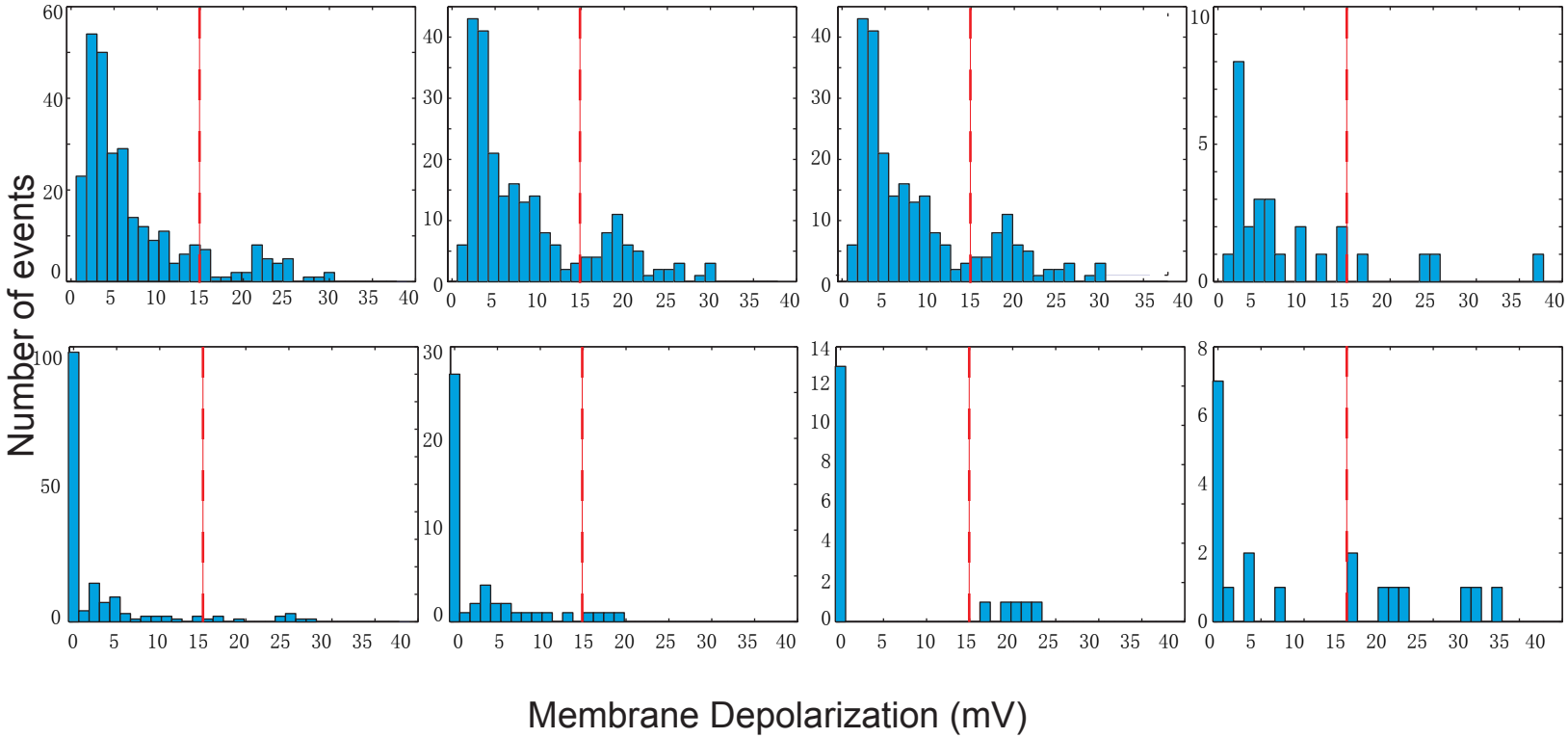

Supplement: Figure S13 — Histograms of membrane potentials at inflection points. Histograms for (A) ChR2-MVIBD-GFP cells (n = 6) and (B) ChR2-GFP cells (n = 8). For each neuron, responses from all laser power stimulations are plotted. Red dashed lines in all cells indicate the threshold (15 mV) for determining axonal versus somatic/dendritic responses. In ChR2-GFP cells there is a small bump in higher membrane potentials, indicating the dendritic component, while this bump almost disappeared in ChR2-MVIBD-GFP cells. In general, there is a trend towards lower membrane potentials in ChR2-MVIBD-GFP cells than in the control ChR2-GFP cells. (PDF) [file pbio.1001021.s013.pdf]

Figure S14. Myosin VI-dependent mechanisms for localization of axonal proteins.

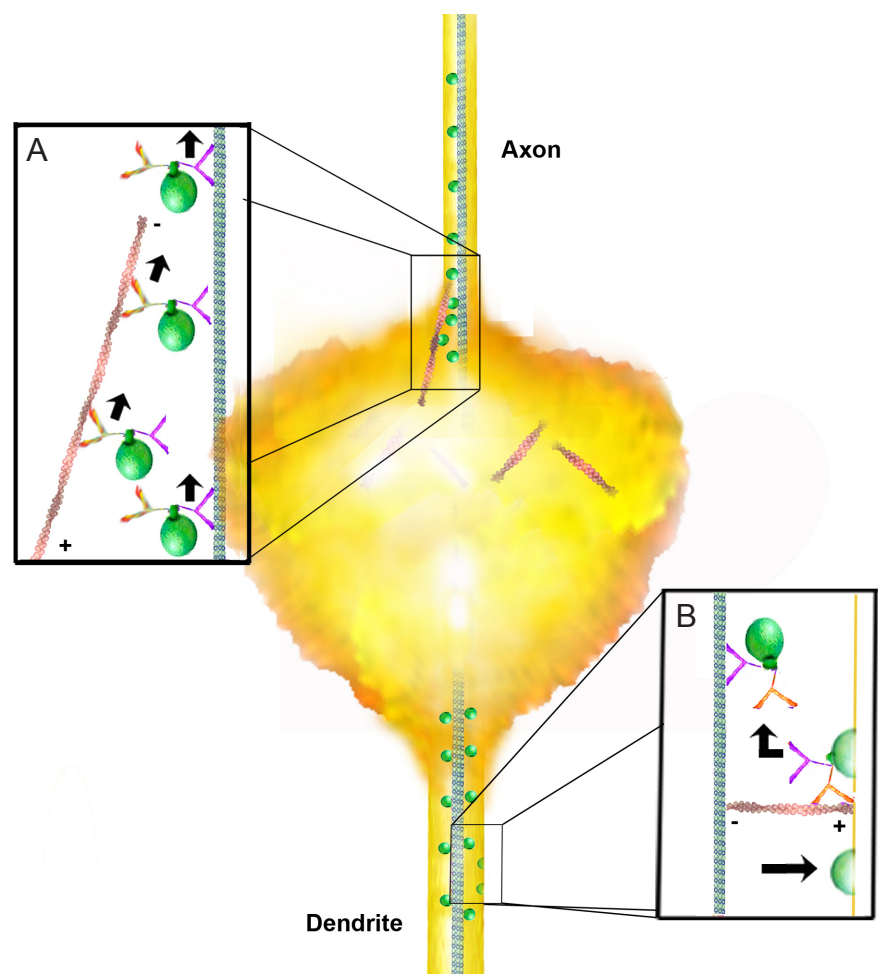

Supplement: Figure S14 — Myosin VI-dependent mechanisms for localization of axonal proteins. Proteins are loaded into vesicles on the surface of the Golgi apparatus and from there are transported either to the axon or to the dendrites by kinesin motors (purple) on microtubules (blue). After the protein sent to the dendrites is deposited on the dendritic surface, it is endocytosed through the actions of Myosin VI (orange, Box B). Proteins loaded into vesicles that proceed to the axon are carried by kinesin motors along microtubules. It is possible that Myosin VI might also guide these vesicles towards the axon by moving along actin filaments (pink) with their plus ends oriented towards the cell body (Box A). In this paper we provide direct evidence that dendrite-specific endocytosis is involved in localization of axonal proteins. Other experiments suggest that a direct mechanism such as the one shown in Box A might also contribute to axonal trafficking, although additional experiments will be required to fully define this mechanism. Note that, for simplicity, only a single microtubule was drawn in the dendrite although there are, in fact, microtubules pointing in both directions. (PDF) [file pbio.1001021.s014.pdf]
