# Supplementary material for: A Role for Myosin VI in the Localization of Axonal Proteins
Source: PLoS Biol. 2011 Mar 1;9(3):e1001021. doi: 10.1371/journal.pbio.1001021 (PMC3046960; doi:10.1371/journal.pbio.1001021)

Figure S8 Neuron expressing MVI siRNA maintains polarized morphology

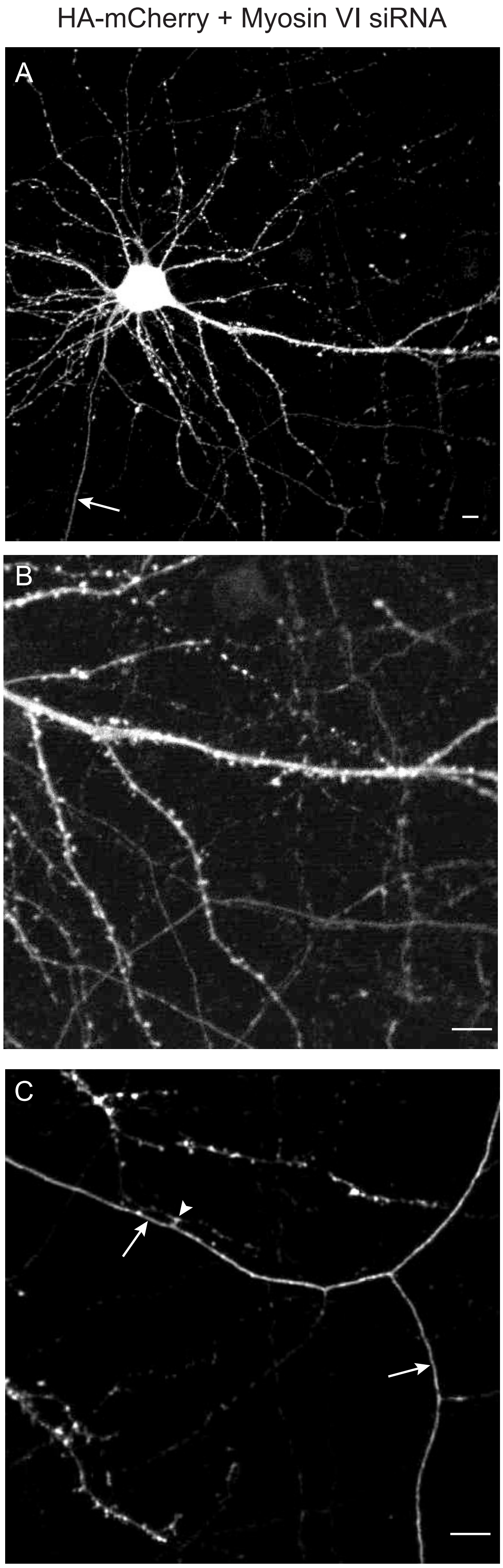

Supplement: Figure S8 — Neurons expressing MVI siRNA maintain polarized morphology. (A) Cortical neuron expressing siRNA against Myosin VI for 14 d. (B) High-power image of cell in (A) showing that dendrites display a tapered morphology and the presence of spines. (C) High-power image of cell in (A) and (B) showing the axon with an untapered morphology and the absence of spines. Note that arrowhead points to an autaptic connection. Arrows point to axon. Scale bars are 10 µm. (PDF) [file pbio.1001021.s008.pdf]
